# Contrasting Effects of Singlet Oxygen and Hydrogen Peroxide on Bacterial Community Composition in a Humic Lake

**DOI:** 10.1371/journal.pone.0092518

**Published:** 2014-03-25

**Authors:** Stefanie P. Glaeser, Bork A. Berghoff, Verena Stratmann, Hans-Peter Grossart, Jens Glaeser

**Affiliations:** 1 Institute for Microbiology and Molecular Biology, Justus-Liebig-University, Giessen, Germany; 2 Institute for Applied Microbiology, Justus-Liebig-University, Giessen, Germany; 3 Department of Cell & Molecular Biology, Uppsala University, Uppsala, Sweden; 4 Leibniz Institute of Freshwater Ecology and Inland Fisheries, Stechlin, Germany; 5 Institute for Biochemistry and Biology, Potsdam University, Potsdam, Germany; Instituto de Biociencias - Universidade de São Paulo, Brazil

## Abstract

Light excitation of humic matter generates reactive oxygen species (ROS) in surface waters of aquatic ecosystems. Abundant ROS generated in humic matter rich lakes include singlet oxygen (^1^O_2_) and hydrogen peroxide (H_2_O_2_). Because these ROS differ in half-life time and toxicity, we compared their effects on microbial activity (^14^C-Leucine incorporation) and bacterial community composition (BCC) in surface waters of humic Lake Grosse Fuchskuhle (North-eastern Germany). For this purpose, experiments with water samples collected from the lake were conducted in July 2006, September 2008 and August 2009. Artificially increased ^1^O_2_ and H_2_O_2_ concentrations inhibited microbial activity in water samples to a similar extent, but the effect of the respective ROS on BCC varied strongly. BCC analysis by 16S rRNA gene clone libraries and RT-PCR DGGE revealed ROS specific changes in relative abundance and activity of major bacterial groups and composition of dominating phylotypes. These changes were consistent in the three experiments performed in different years. The relative abundance of *Polynucleobacter necessarius, Limnohabitans*-related phylotypes (*Betaproteobacteria*), and *Novosphingobium acidiphilum* (*Alphaproteobacteria*) increased or was not affected by photo-sensitized ^1^O_2_ exposure, but decreased after H_2_O_2_ exposure. The opposite pattern was found for *Actinobacteria* of the freshwater AcI-B cluster which were highly sensitive to ^1^O_2_ but not to H_2_O_2_ exposure. Furthermore, group-specific RT-PCR DGGE analysis revealed that particle-attached *P. necessarius* and *Limnohabitans-*related phylotypes exhibit higher resistance to ^1^O_2_ exposure compared to free-living populations. These results imply that ^1^O_2_ acts as a factor in niche separation of closely affiliated *Polynucleobacter* and *Limnohabitans*-related phylotypes. Consequently, oxidative stress caused by photochemical ROS generation should be regarded as an environmental variable determining abundance, activity, and phylotype composition of environmentally relevant bacterial groups, in particular in illuminated and humic matter rich waters.

## Introduction

Dissolved organic matter (DOM) is the major carbon and energy source for heterotrophic bacteria in aquatic ecosystems [1]. Humic lakes are characterized by a high content of allochthonous DOM with recalcitrant high-molecular-weight poly-aromatic compounds. Photochemical transformations of these compounds generate low-molecular-weight substances and thereby stimulate microbial activity and growth [2], [3]. On the other hand, photochemical processes lead to inhibitory effects including (i) photo-oxidation and (ii) transformation of labile compounds [4], [5] as well as (iii) generation of reactive intermediates such as reactive oxygen species (ROS) [6], [7], [8]. ROS generation in aquatic ecosystems occurs by light excitation of DOM, in particular humic matter, and subsequent formation of triplet excited states in poly-aromatic compounds [8]. Light-excited DOM transfers energy or electrons to molecular oxygen. Thereby, the transfer of energy generates singlet oxygen (^1^O_2_) and the incomplete reduction of oxygen leads to the formation of hydrogen peroxide (H_2_O_2_) and other ROS. Recent experiments strongly suggest that distinct structures in humic matter are linked to the formation of ^1^O_2_ or H_2_O_2_ [9] and that the reaction of ^1^O_2_ with DOM generates small amounts of H_2_O_2_ [9], [10].

Effects of photochemically altered DOM on microorganisms were mainly investigated via inoculation of pre-irradiated DOM with natural microbial assemblages [11], [12] including studies, which examined the effect of substrate availability on bacterial community composition (BCC) [13], [14], [15]. In a recent study, we showed that ^1^O_2_ has the potential to inhibit typical freshwater bacterial species and consequently affect BCC [16]. Singlet oxygen is highly reactive, exhibits a half-life time in water of ∼3.5 μs [17], and causes cell damage by oxidation of lipids, nucleic acids, and proteins [18], [19]. In contrast, H_2_O_2_ has a half-life time of up to 8 hours in freshwater [20]. Moreover, H_2_O_2_ diffuses through biological membranes and mainly reacts with iron-sulphur clusters leading to subsequent intracellular hydroxyl radical formation and damage of biomolecules [21]. Hence, potentials for cell damage caused by ^1^O_2_ and H_2_O_2_ differ substantially.

In a previous study, short and long term effects of ^1^O_2_ on BCC were investigated [16]. The present study compares effects of increased ^1^O_2_ and H_2_O_2_ concentrations on BCC and includes experiments on the activity of heterotrophic bacteria in the surface water of the humic matter rich south-west (SW) basin of Lake Grosse Fuchskuhle (North-eastern Germany). The experiments were designed to elucidate differences in sensitivity of dominating bacterial phylotypes towards naturally occurring ROS of different toxicity. We tested the following hypotheses: i) ^1^O_2_ and H_2_O_2_ exposure elicit specific changes in microbial activity and BCC and ii) ROS-induced changes differ between particle-attached and free-living bacterial communities. Investigation of the latter hypothesis is of particular importance since a higher generation of ^1^O_2_ has been observed in particles compared to the ambient water in humic matter rich ecosystems [22], [23].

## Results

### 1O_2_ and H_2_O_2_ C°ncentrations in Surface Water Samples

**Figure 1:**
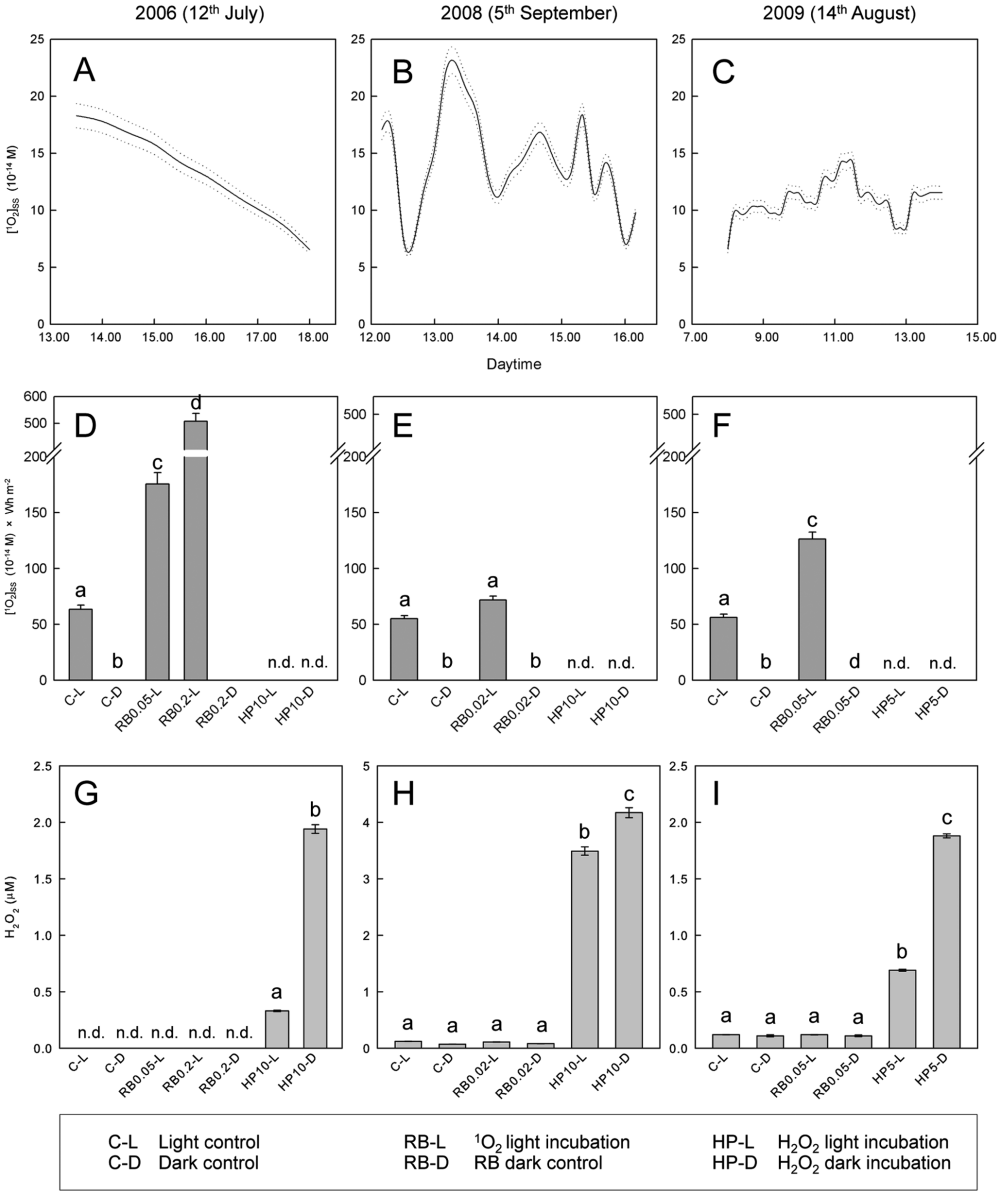
Formation of ^1^O_2_ and H_2_O_2_ during experiments in 2006, 2008 and 2009. Kinetics of [^1^O_2_]_SS_ in the surface water layer (A–C) were calculated from the rate of furfuryl alcohol decay and the light intensity according to Haag and Hoigne (1986). The formation of ^1^O_2_ largely depends on the light intensity (Table S1) and hence [^1^O_2_]_SS_ kinetics depend on the weather conditions. A. 12^nd^ July 2006: a clear sky during the afternoon led to a steady decrease in [^1^O_2_]_SS_ concentrations from noon to late afternoon. B. 5^th^ September 2008: a cloudy sky during the afternoon caused fluctuation in [^1^O_2_]_SS_ concentrations. C. 14^th^ August 2009: a slightly overcasted sky during the whole day led to reduced fluctuations in [^1^O_2_]_SS_ concentrations compared to 2008. Values for solar radiation and rainfall within 30 days prior to the experiments were similar (Fig. S9) and hence all three experiments were conducted under comparable environmental situations. The addition of Rose Bengal (RB) increased the formation of ^1^O_2_ (D–F). D. 2.8 -fold for RB0.05-L and 8-fold for RB0.2-L in 2006, E. 1.3-fold in 2008, and F. 1.9-fold in 2009. Hydrogen peroxide concentrations were analysed in all samples at the end of the experiments (G–H). G. and H. 10 μM H_2_O_2_ were added in 2006 and 2008, respectively. I. 5 μM H_2_O_2_ were added in 2009. Numbers at RB and HP on the x-axis labels correspond to μM concentrations of RB or H_2_O_2_. Please note the different scale in panel H compared to panels G and I. n.d.: not determined. An overview of the abbreviations used for the experimental setups is given in the box at the bottom of the Figure. C–L/D: Light and dark control incubations, RB-L: Light incubation with increased [^1^O_2_]_SS_, RB-D: Dark control for RB, HP-L/D: Light and dark incubations with H_2_O_2_. Dotted lines in A–C and error bars in D–F represent the standard deviation of the FFA method where three distinct water samples were used to determine sample specific [^1^O_2_]_SS_ concentrations. Error bars in G–H indicate the standard deviation of three analysed samples. Different letters at the top of the bars depict statistically significant differences (with p≤0.001) between values as determined by one-way ANOVA followed by pair-wise multiple comparison analysis with the Tukey’s test performed in Sigma Stat v. 2.0 (Systat Software). The same letters indicate that depicted values are not significantly different to each other.

Three sets of *in situ* experiments were performed in July 2006, September 2008 and August 2009. For each experiment day, ^1^O_2_ steady state [^1^O_2_]_SS_ concentrations and H_2_O_2_ formation were determined, because variations in sunlight intensity and in concentration of dissolved organic carbon (DOC) were observed (Table 1). By applying the furfuryl alcohol (FFA) method [24] we observed similar *in situ* [^1^O_2_]_SS_ concentrations on all three experiment days that were in the range of 11.2 to 14.1×10^−14^ M in the surface water layer of the lake (Table 1). The kinetics of ^1^O_2_ formation differed between experiment days (Fig. 1A–C), but the dose of ^1^O_2_ exposure was very similar and ranged from 56.2 to 63.5×10^−14^ M Wh m^−2^ (C-Ls; Fig. 1D–F). Hydrogen peroxide concentrations were low in all water samples. During diurnal cycle studies ∼50 nM were detected on 11^th^ July 2006 (data not shown), but in 2008 and 2009, H_2_O_2_ concentrations were in the range of 70 to 120 nM (Fig. 1H and I).

**Table 1:** Selected environmental parameters on experiment days in 2006, 2008 and 2009.

Parameter	Sample		
	2006 (12^nd^ July)	2008 (5^th^ September)	2009 (14^th^ August)
**DOC (mg C L^−1^)**	23.3±1.8	34.0±0.1	28.4±1.1
**Average light intensity** **(W m^−2^)**	570	445	557
***In situ*** ** [^1^O_2_]_SS_ (10^−14^ M)**	14.1±0.8	11.8±0.01	11.2
***In situ*** ** H_2_O_2_ (nM)**	n.d.	120±2.5	120±1.42

DOC concentration, average light intensity and subsequent [^1^O_2_]_SS_ and H_2_O_2_ concentrations slightly differed between experiment days of the three studied years.

n.d.: not determined.

Environmental conditions with respect to ROS concentrations may have varied throughout the years. In order to ensure that the reactivity of natural organic matter (NOM) was similar on each experiment day, 0.22 μm filtered water samples were further analysed (Materials S1). Normalization of ROS formation to mg DOC L^−1^ revealed that the specific ^1^O_2_ formation was similar between the experiment days. In contrast, the specific H_2_O_2_ formation was higher in 2009 compared to 2006 and 2008 (Table S1). Large variations of *in situ* ^1^O_2_ formation were not observed. In contrast, for H_2_O_2_ an up to 4–5 fold variation in formation rate was detected (Table S1), but concentrations measured in lake water samples remained similar (Fig. 1H and I).

### Modification of ^1^O_2_ and H_2_O_2_ Concentrations

**Figure 2:**
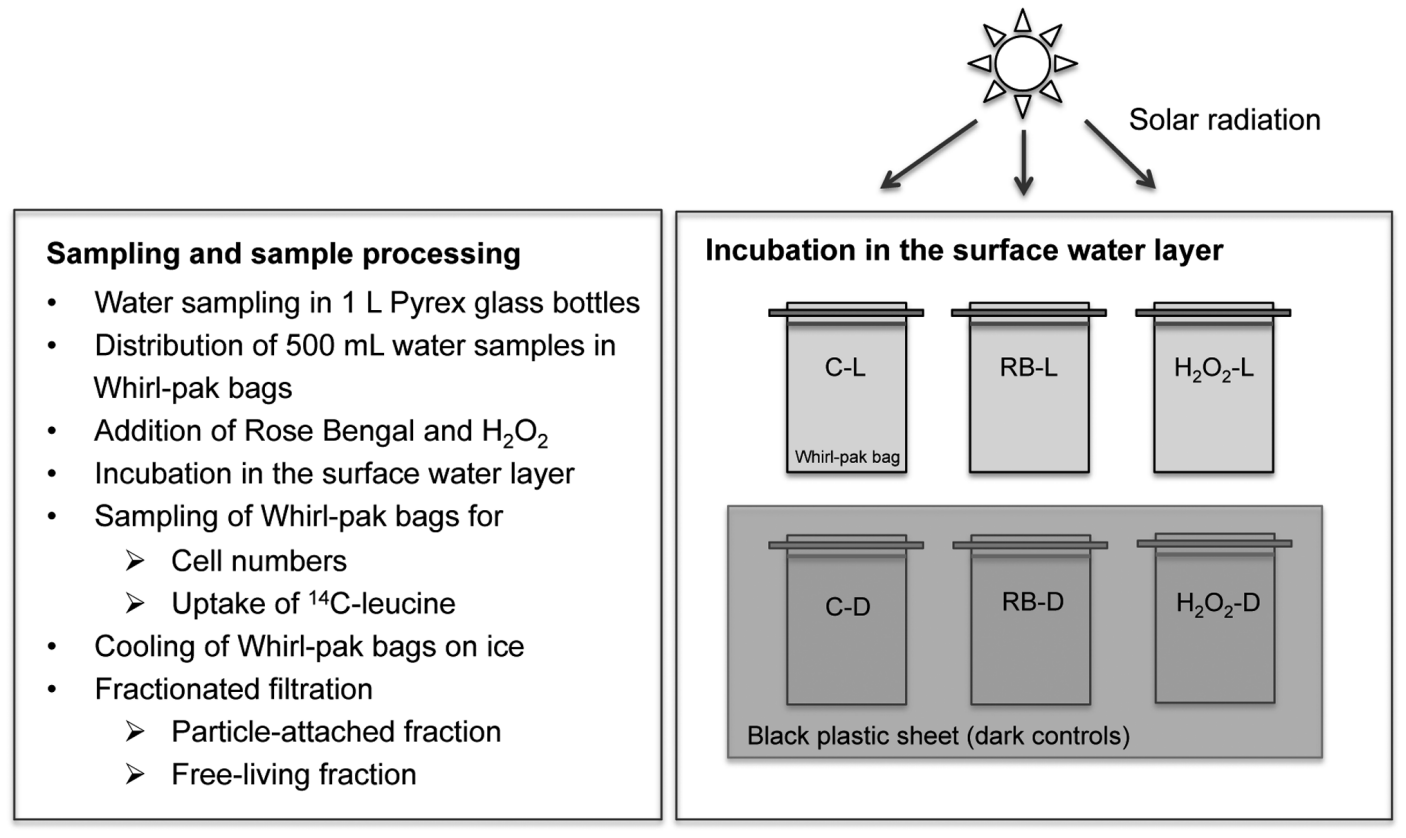
Design of field experiments. Field experiments performed in 2006, 2008 and 2009 followed the same experimental outline as displayed in the flow chart. Whirl-pak bags were incubated in the surface water layer on large metal racks after addition of Rose Bengal and H_2_O_2_. Dark controls were covered with a black plastic sheet to avoid exposure to solar radiation. Abbreviations are given in Fig. 1.

All *in situ* experiments performed in the summers of 2006, 2008 and 2009 were designed to test whether effects of increased ^1^O_2_ and H_2_O_2_ concentrations consistently differ in surface waters (hypothesis i). Respective field experiments (Fig. 2) were performed by obtaining water samples from the lake. Increased environmental ROS levels, particularly of H_2_O_2_, were obtained by adding the photosensitizer Rose Bengal (RB), a poly-aromatic compound that specifically generates ^1^O_2_ in the presence of light and oxygen or by H_2_O_2_ addition.

Concentrations of ^1^O_2_ increased by 1.3 to 8-fold in light incubations after RB addition (Fig. 1D–F). Addition of 5 μM H_2_O_2_ in 2009 or 10 μM in 2006 and 2008 represented an increase in H_2_O_2_ concentrations by ∼45 to 200-fold, respectively. In experiments with H_2_O_2_ addition, the concentrations decreased during the time of incubation and ranged between 0.25 and 4.2 μM at the end of the experiments. Concentrations of H_2_O_2_ were lower in light incubations compared to dark controls (Fig. 1G–I) and H_2_O_2_ end concentrations were ∼3 to 33-fold higher compared to the non-amended controls. This notion is in line with the high capacity for H_2_O_2_ degradation found for humic matter rich water samples of the SW compartment (Materials S1).

### Microbial Activity is Hampered by ROS Exposure

**Figure 3:**
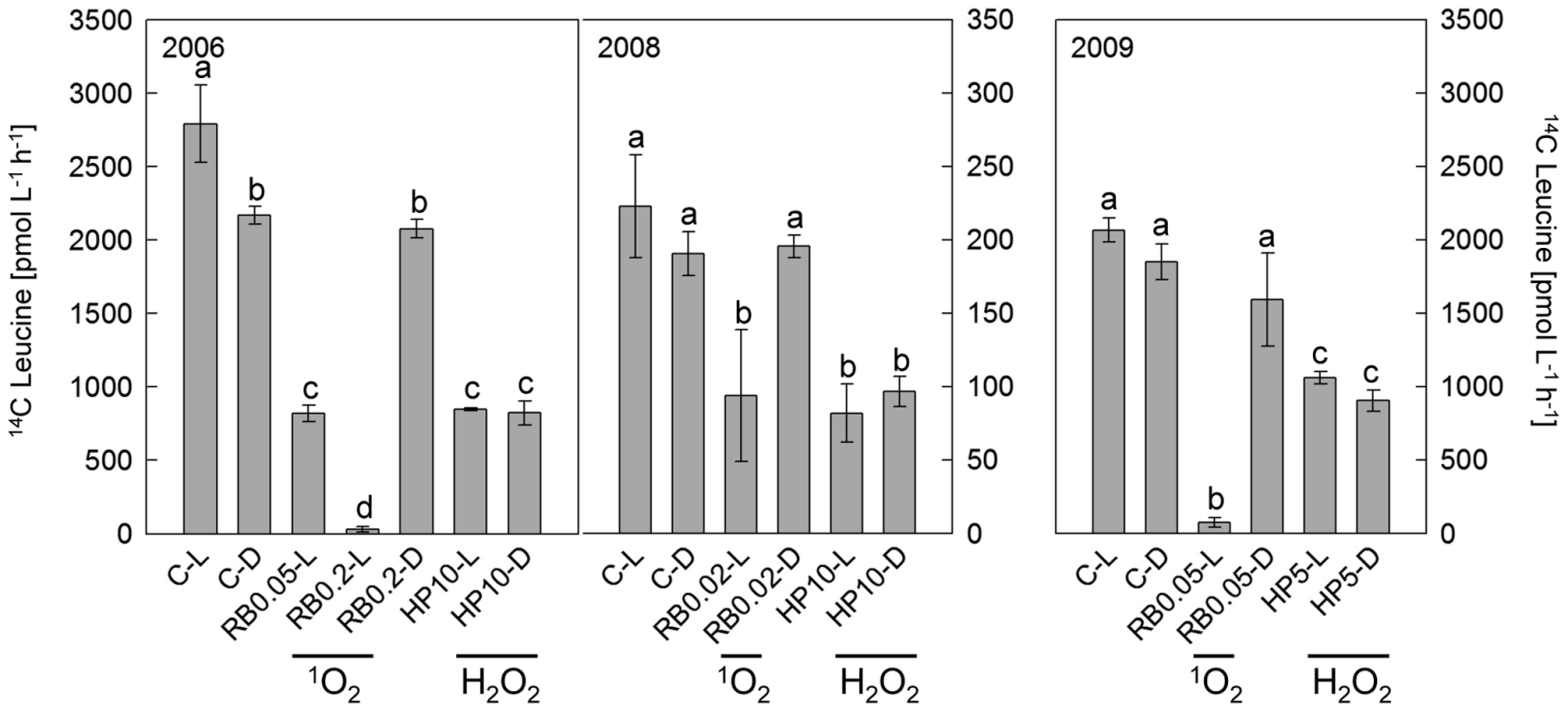
Activity of heterotrophic microorganisms after ^1^O_2_ and H_2_O_2_ exposure. Microbial activity was measured as leucine incorporation during 1(with p≤0.001) between values as determined by one-way ANOVA followed by pair-wise multiple comparison analysis with the Tukey’s test performed in Sigma Stat v. 2.0 (Systat Software). The same letters indicate that depicted values are not significantly different to each other. Tests were done separately for each year. Abbreviations are given in Fig. 1.

Activity of heterotrophic microorganisms, assessed by ^14^C-Leucine incorporation, was highest in the light controls (C–L) reaching 2800, 223, and 2100 pmol leucine L^−1^ h^−1^ in 2006, 2008, and 2009, respectively (Fig. 3). In 2006, microbial activity was significantly higher in the light than in the dark control. A similar trend occurred in 2008 and 2009, but it was not statistically significant. In all experiments, increased ROS levels caused inhibition of microbial activity. Precisely, generation of ^1^O_2_ (RB0.05-L) and addition of H_2_O_2_ (HP10-L/D) decreased microbial activity to 30% of that in the respective C–L in 2006. Similar treatments caused a decrease to 43% in 2008. In 2009, the addition of 5 μM H_2_O_2_ in light and dark treatments (HP5-L/D) resulted in a decrease of microbial activity to 51 and 44% of that in the respective C–L. Singlet oxygen generation in RB0.2-L in 2006 and RB0.05-L in 2009 decreased microbial activity to below 5% of the respective C–L. In 2009, particle-attached and free-living bacteria were assessed separately to investigate differences in their potential to incorporate leucine. In controls, particle-attached bacteria incorporated 2.3 to 2.6-fold more leucine than free-living bacteria. Exposure to ROS decreased the activity of both fractions to the same extent (Fig. S1), indicating an overall similar sensitivity of the microbial community to ROS exposure.

Significant changes in cell numbers were not correlated with ROS exposure, except for the ^1^O_2_ exposure in 2008 (Fig. S2). As observed in earlier experiments [16] increased numbers of micrococcoid cells were responsible for elevated total cell numbers (Table S2).

Different concentrations of RB and H_2_O_2_ were used on the three experiment days (Fig. 3). Overall, we aimed for a similar inhibition of microbial activity by ^1^O_2_ and H_2_O_2_ in order to enable a direct comparison of changes in BCC within each experiment. Therefore, several RB concentrations were tested in 2006 and 2008 (data not shown) and for further analysis only those treatments were chosen which showed a similar inhibition of ^14^C-Leucine incorporation.

### Relative Abundance of Bacterial Groups After ^1^O_2_ and H_2_O_2_ Exposure

**Figure 4:**
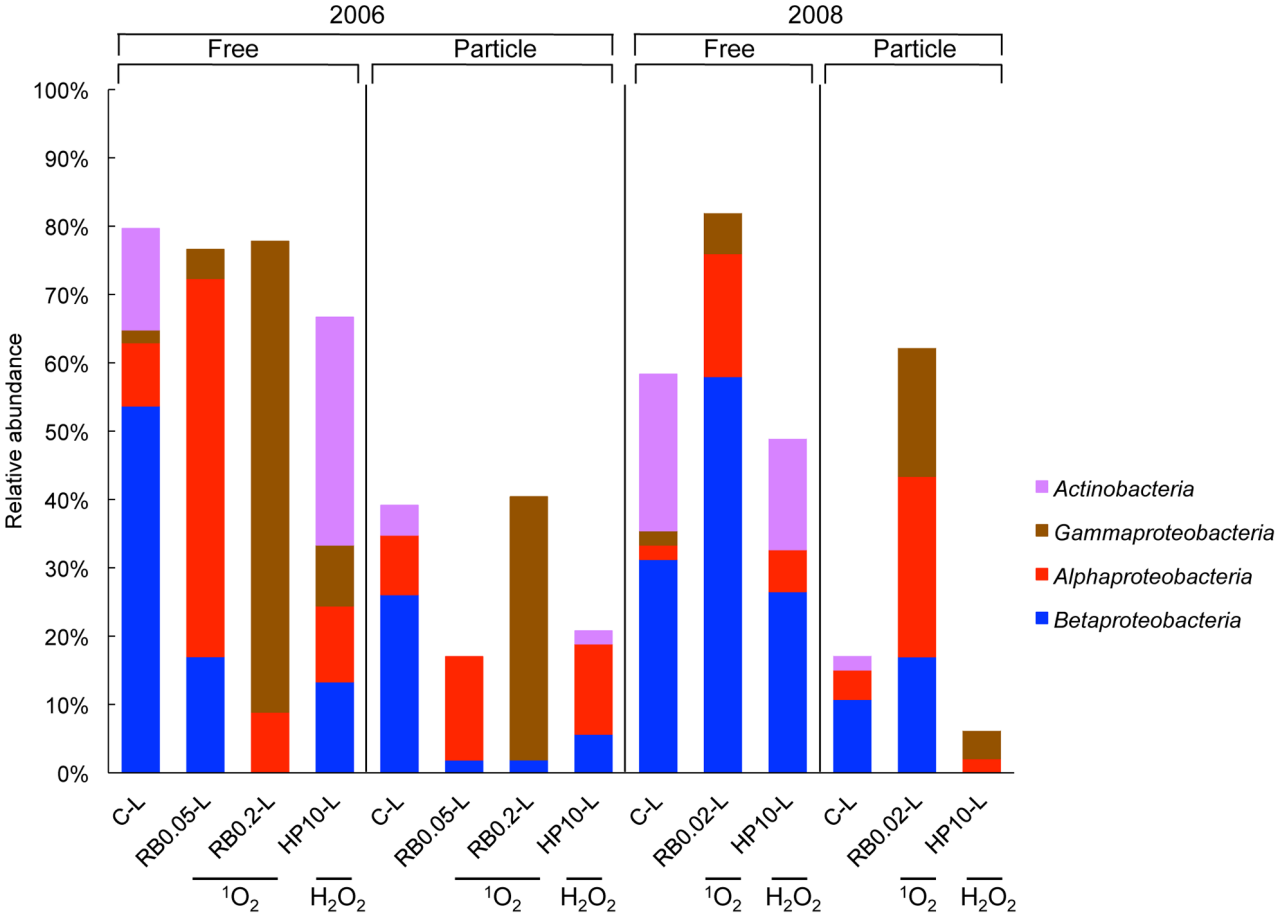
Relative abundance of major bacterial groups. 16S rRNA gene clone libraries generated with universal bacterial primers obtained from free-living (0.22–8 μm in 2006 and 0.22–5 μm in 2008) and particle-attached (>8 or >5 μm, respectively) bacterial fractions after ^1^O_2_ and H_2_O_2_ exposure. Clone libraries were generated for control (C-L), ^1^O_2_ (RB-L) and H_2_O_2_ (HP-L) light treatments of *in situ* experiments 2006 and 2008. The relative abundance represents fractions (%) of all investigated clones of each clone library. For abbreviations see Fig. 1. Colours indicate the phylogenetic affiliation: *Actinobacteria* (purple), *Gammaproteobacteria* (brown), *Alphaproteobacteria* (red), and *Betaproteobacteria* (blue).

Clone libraries of free-living bacterial fractions in light controls (C-Ls) in 2006 and 2008 (Fig. 4) were dominated by Betaproteobacteria (54 and 31%), followed by Actinobacteria (15 and 23%) and Alphaproteobacteria (9 and 2%). In the respective particle-attached fractions, Betaproteobacteria (26 and 10%) and Bacteroidetes (11 and 13%, Table 2) dominated, followed by Alphaproteobacteria (9 and 4%), and Actinobacteria (4 and 2%). In both years, less abundant groups including Firmicutes, Chlorobii, Verucomicrobia, and Acidobacteria represented only 4 to 6% of free-living as well as 6 to 13% of particle-attached bacterial fractions (Table 2). In 2006 and 2008, chloroplast sequences accounted for 2 and 15% of free-living or 24 and 31% of the particle-attached fractions, respectively.

**Table 2:** Relative abundance and phylogenetic affiliation of sequenced phylotypes.

		2006								2008											
		0.22–8 μm				>8 μm				0.22–5 μm			>5 μm								
	OTUs	C-L	RB0.05-L	RB0.2-L	HP10-L	C-L	RB0.05-L	RB0.2-L	HP10-L	C-L	RB0.02-L	HP10-L	C-L	RB0.02-L	HP10-L	Freshwatercluster	RDP Naive Bayesian rRNAClassifier		BLAST results		
*β-Proteobacteria*	**1**	31	17		4	9	2	2	4	13	30	8	4	2		bet II, PnecC	*Burkholderiaceae*	100%	99%	*P. necessarius*QLW-P1-DMWA-1T	CP000655
	**2**				4	2						6				bet II, PnecA	*Burkholderiaceae*	100%	97%	*P. acidophobus*MWH-PoolGreenA3	FM208180
	**3**	2			2	13			2	13	20	4	2	2		bet I, Lhab-A4	*Comamonadaceae*	98%	99%	Lake GrosseFuchskuhle clone FNE11-10	DQ501302
	**4**	19			2	2				2	2	2				bet IV, RDP18A09	*Methylophilaceae*	100%	98%	Parker river clonePRD18A09	AY947994
	**5**													4		bet III, betIII-A1	*Alcaligenaceae*	100%	99%	Grosse Lacke isolateQLW-p2DMWB-4	AJ938031
	**6**	2								4	2			4		bet I, Lhab-A4	*Comamonadaceae*	100%	97%	Lake IJssel clone Stal-17	AJ416187
	**7**										4	6	4	6		bet VII	*Oxalobacteraceae*	100%	97%	Lake Grosse Fuchskuhleclone NE45	AJ575695
*α-Proteobacteria*	**8**	9	36	2	4	7	4			2	16	6		13		alf IV-A, Novo-A1	*Sphingomonadaceae*	100%	99%	*N. acidiphilum*FSW06-204dT	EU336977
	**9**		19				11		2								*Bradyrhizobiaceae*	15%	96%	Lake Pohlseeclone Hv_38	EF667926
	**10**			7													*Hyphomicrobiaceae*	36%	90%	*Mesorhizobium* sp.CCBAU 33182	GU433452
	**11**										2		4	9	2	alf II	*Caulobacteraceae*	100%	97%	Adriondack lake cloneADK-BTe02-51	EF520395
	**12**													4			*Hyphomicrobiaceae*	46%	98%	*Rhodomicrobium vannielii*E.Y. 33T	M34127
	**13**				7				2							alf I, alf I-B1	*Beijerinckiaceae*	69%	98%	Lake Grosse Fuchskuhleisolate FSW06-301	FJ798303
	**14**					2			9							alf VIII	*Acetobacteraceae*	100%	95%	*Asaia lannaensis* BCC 15733T	AB286050
*γ-Proteobacteria*	**15**	2		58	2			13								close to gam III	*Methylococcaceae*	100%	95%	Hypertrophic freshwater lakeclone ML-9-70.2	DQ520192
	**16**			7	7			15									*Legionellaceae*	100%	96%	*Legionella longbeachae*ATCC 33484	AY444741
	**17**		4	4				6									*Legionellaceae*	99%	92%	*Legionella impletisoli*OA1-1T	AB233209
	**18**							4									*Ectothiorhodospiraceae*	70%	96%	Activated sludge cloneAS1o9	AJ514448
	**19**									2	6			19	4	gam I	*Methylococcaceae*	100%	96%	*Methylomonas rubra*NCIMB 11913	AF304194
*Actinobacteria*	**20**	15			33	4			2	23		16	2			acI-B, scB-3	*Microbacteriaceae*	45%	99%	Lake Grosse Fuchskuhleclone FSW11-16	DQ316348
*Firmicutes*	**21**		2	4		2	17	27	8								*Paenibacillaceae*	95%	93%	*Paenibacillus polymyxa* SC2	CP002213
*Chlorobii*	**22**								4		2	2	9	17	17		*Chlorobiaceae*	100%	98%	*Pelodictyon* *phaeoclathratiforme*BU-1T	CP001110
*Bacteroidetes*	**23**	2				11			4	4			13		4		*Chitinophagaceae*	100%	98%	Lake Grosse Fuchskuhleclone FukuS59	AJ290042
	**24**								4								*Sphingobacteriaceae*	100%	96%	Tatachia forest soil cloneTSC56	EU359966
*Verucomicrobia*	**25**													4	2		Subdivision5	78%	97%	Lake Kinneret sedimentclone d0-26	AM409824
*Acidobacteria*	**26**					4				2	8	6	4	2			*Holophagaceae*	100%	96%	*Geotrix fermentans*ATCC 700665	U41563
Chloroplasts	**27**		13	2		7	55	2	13								*Bacillariophyta*	87%	97%	Parker river clonePRD18F11	AY948053
	**28**						4		2						8		*Chlorarachniophyceae*	40%	93%	Parker river clonePRD18D01	AY948021
	**29**	2			20	4				15		12	2		6		*Chlorarachniophyceae*	68%	93%	Adriondack lake cloneADK-HDe02-54	EF520517
	**30**					9			8				17		29		*Cryptomonadaceae*	100%	94%	Adirondack lake cloneADK-SGh02-76	EF520521
	**31**					4			8				13	2	17		*Cryptomonadaceae*	100%	98%	Parker river clonePRD18E12	AY948043
	**32**								8								*Chlorophyta*	100%	91%	*Polytoma oviforme*cloroplast	AF374188
Rare OTUs (%)*		6	2	2	7	7	2	8		4	4	4	2								
Single OTUs (%)#		11	6	13	7	13	6	23	23	17	4	27	23	13	10						
Total No. ofclones		54	47	45	45	46	53	52	53	48	50	49	47	53	48						
**Coverage (%)**		**83**	**91**	**84**	**87**	**80**	**92**	**69**	**77**	**79**	**92**	**69**	**74**	**87**	**90**						

*Rare OTUs: OTUs that occur only once in one clone library;

# Single OTUs: OTUs that occur only once in at least two clone libraries.

Exposure to ^1^O_2_ and H_2_O_2_ induced specific shifts in BCC. Increased ^1^O_2_ exposure led to the disappearance of Actinobacteria and Bacteroidetes in both free-living and particle-attached fractions, whereas the effects on Beta-, Alpha-, and Gammaproteobacteria as well as Firmicutes depended on ^1^O_2_ dose and bacterial fraction (Fig. 4, Table 2). In 2006, a 2.8-fold increased ^1^O_2_ exposure decreased Betaproteobacteria by 37 and 24% in the free-living and particle-attached fraction, respectively. In contrast, Alphaproteobacteria increased by 46% in the free-living and by 6% in the particle-attached fraction, whereas Firmicutes increased by 15% only in the particle-attached fraction (Table 2). After an 8-fold increased ^1^O_2_ exposure, Gammaproteobacteria dominated and accounted for 69 and 38% of the free-living and particle-attached fraction, respectively. In contrast, Alphaproteobacteria disappeared in the particle-attached fraction, but did not change in the free-living one. Firmicutes strongly increased by 25% exclusively in the particle-attached fraction (Table 2). The much lower 1.3-fold elevated ^1^O_2_ exposure in 2008 increased Betaproteobacteria by 27 and 7% in the free-living and particle-attached fraction, respectively. In both fractions, Alphaproteobacteria increased by 16 and 22%, and Gammaproteobacteria by 4 and 19%.

After H_2_O_2_ exposure, BCC changed in a very different manner. The abundance of free-living Betaproteobacteria decreased by 41 and 4% in 2006 and 2008, but in both years they remained highly abundant (Fig. 4). Particle-attached Betaproteobacteria decreased after H_2_O_2_ exposure by 20% in 2006, and were not detected in 2008. The change in relative abundance of free-living Actinobacteria varied between an 18% increase (2006) and a 7% decrease (2008), but negative effects were less pronounced than after exposure to ^1^O_2_. Actinobacteria remained highly abundant and the relative abundance of further bacterial groups only slightly changed after H_2_O_2_ exposure (Table 2).

### Changes in the Overall Bacterial Diversity by Clone Library Analysis

The coverage of the individual clone libraries ranged between 69 and 92%, with a mean coverage value of 82.4% (Table 1). Rarefaction analysis showed that rarefaction curves generated for each clone library did not reach complete saturation by a number of approx. 50 clones for each investigated clone library (Fig. S3). The lack of saturation was mainly due to single and rare OTUs, which ranged between 8 to 31%. The focus of our study, however, was on investigating ROS-induced changes in relative abundance of the most prominent freshwater bacterial groups or species. Therefore, such single and rare OTUs were not investigated by sequence analysis and our clone library analyses did not aim to cover the overall diversity within each treatment. The number of investigated OTUs was sufficient to depict distinct differences in phylotype abundance after increased ^1^O_2_ and H_2_O_2_ exposure. Especially for free-living bacteria, rarefaction curves were closer to saturation after exposure with 0.05 μM RB in the light (^1^O_2_ treatments) in 2006 and 2008, respectively (Fig. S3). This finding indicates that bacterial diversity after ^1^O_2_ treatments are lower than in C–L and H_2_O_2_ light treatments for experiments in 2006 and 2008.

### Effects of ^1^O_2_ and H_2_O_2_ Exposure on Predominant Bacterial Phylotypes

**Figure 5:**
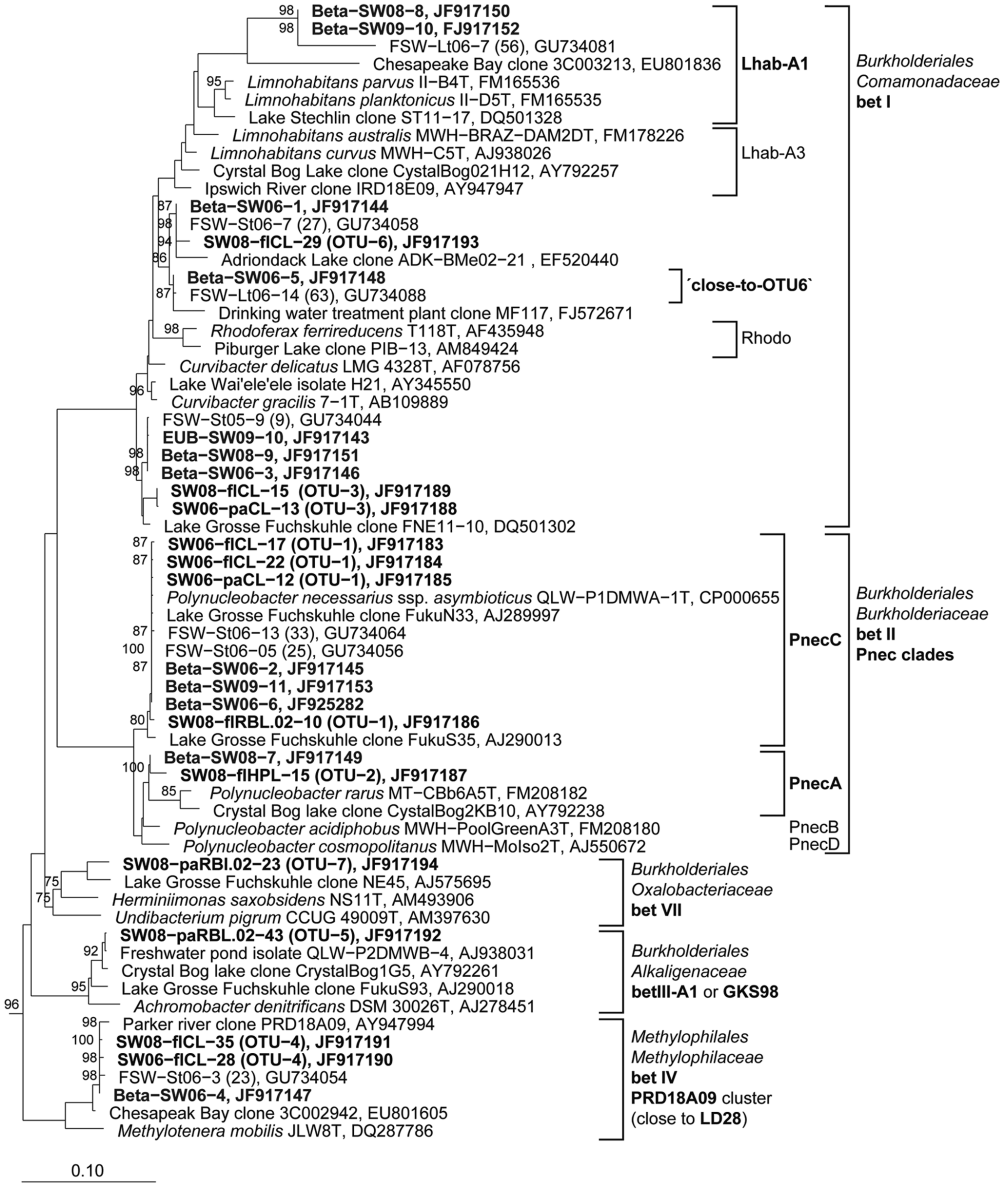
Phylogenetic affiliation of 16S rRNA gene sequences representing OTUs and DGGE bands to the *Betaproteobacteria.* Maximum likelihood trees showing the phylogenetic affiliation of OTU and DGGE band sequences to the *Betaproteobacteria.* Sequences obtained from DGGE bands are depicted in bold letters. Numbers at roots represent bootstrap values (≥70%) of 100 re-samplings. Scale bars: 0.1 nucleotide substitutions per site. Sequences representing OTUs are assigned as follows: SW: South West basin, 06, 08: year of *in situ* experiment in 2006 or 2008, fl: free-living bacteria, pa: particle-attached bacteria. Sequences signed with EUB, Beta, or Actino are from *Bacteria, Betaproteobacteria*, or *Actinobacteria*-specific RT-PCR DGGE bands, respectively.

**Figure 6:**
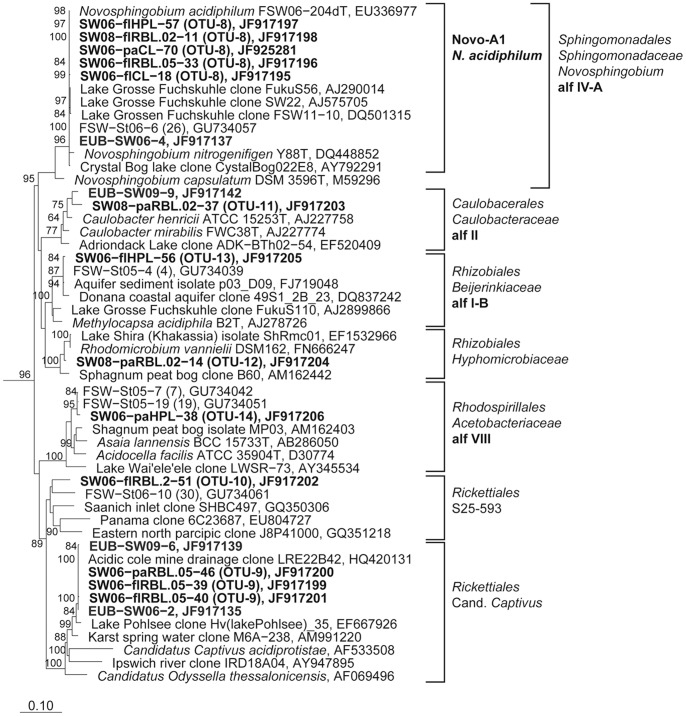
Phylogenetic affiliation of 16S rRNA gene sequences representing OTUs and DGGE bands to the *Alphaproteobacteria.* Maximum likelihood trees showing the phylogenetic affiliation of OTU and DGGE band sequences to the *Alphaproteobacteria.* Details and abbreviations are indicated in the legend to Figure 5.

**Figure 7:**
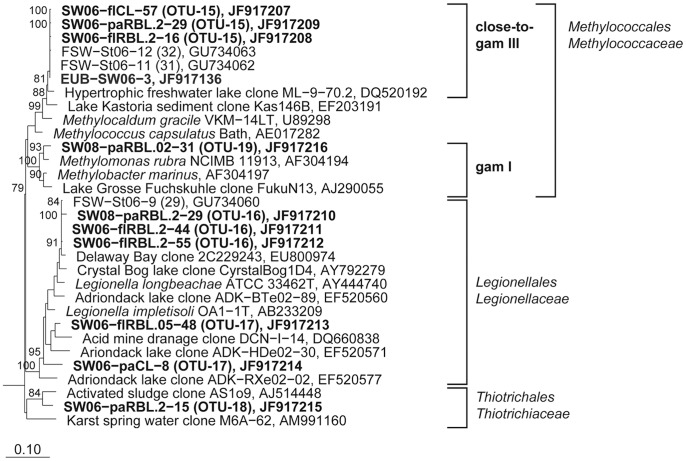
Phylogenetic affiliation of 16S rRNA gene sequences representing OTUs and DGGE bands to the *Gammaproteobacteria.* Maximum likelihood trees showing the phylogenetic affiliation of OTU and DGGE band sequences to the *Gammaproteobacteria.* Details and abbreviations are indicated in the legend to Figure 5.

**Figure 8:**
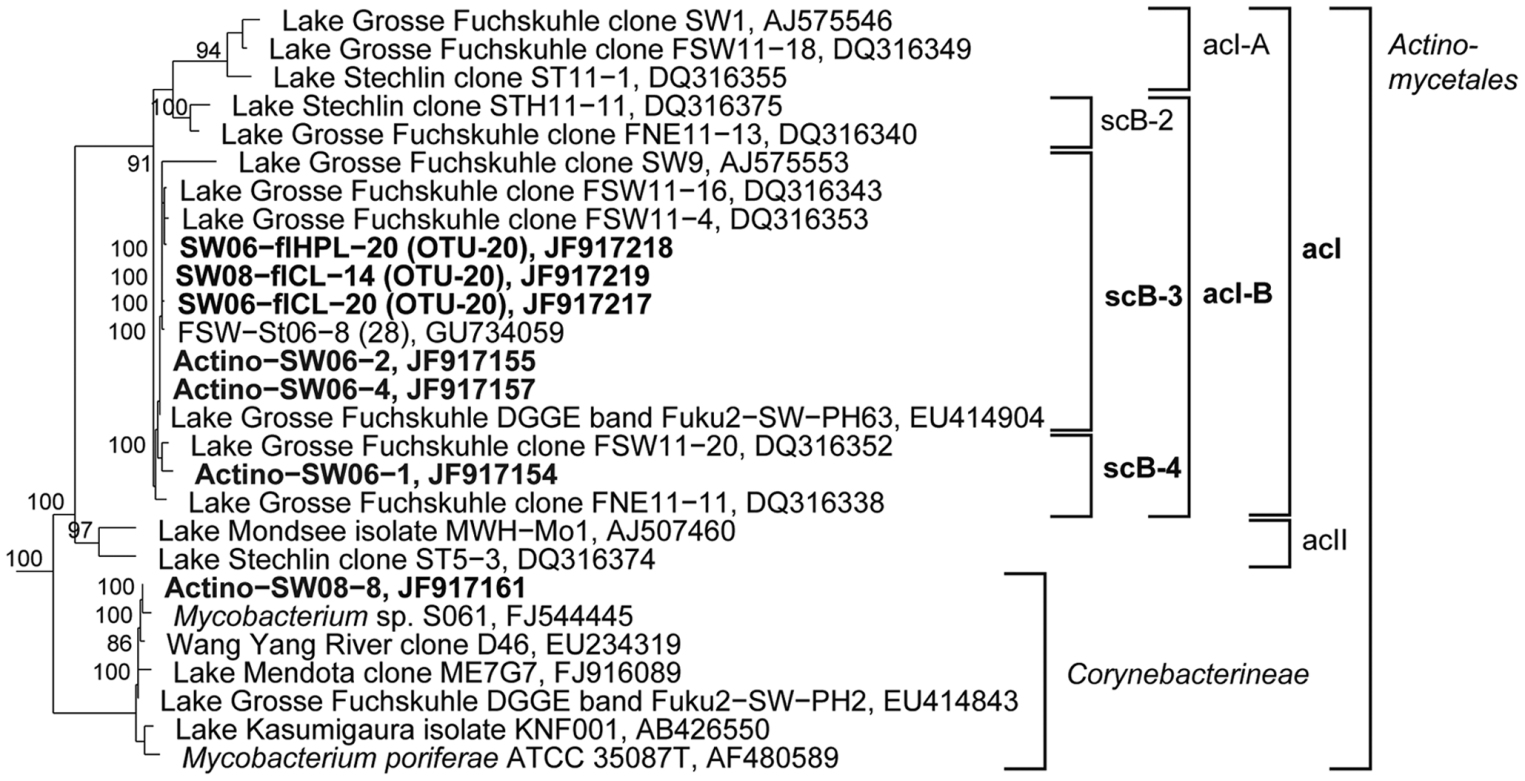
Phylogenetic affiliation of 16S rRNA gene sequences representing OTUs and DGGE bands to the Actinobacteria. Maximum likelihood trees showing the phylogenetic affiliation of OTU and DGGE band sequences to the Actinobacteria. Details and abbreviations are indicated in the legend to Figure 5.

**Figure 9:**
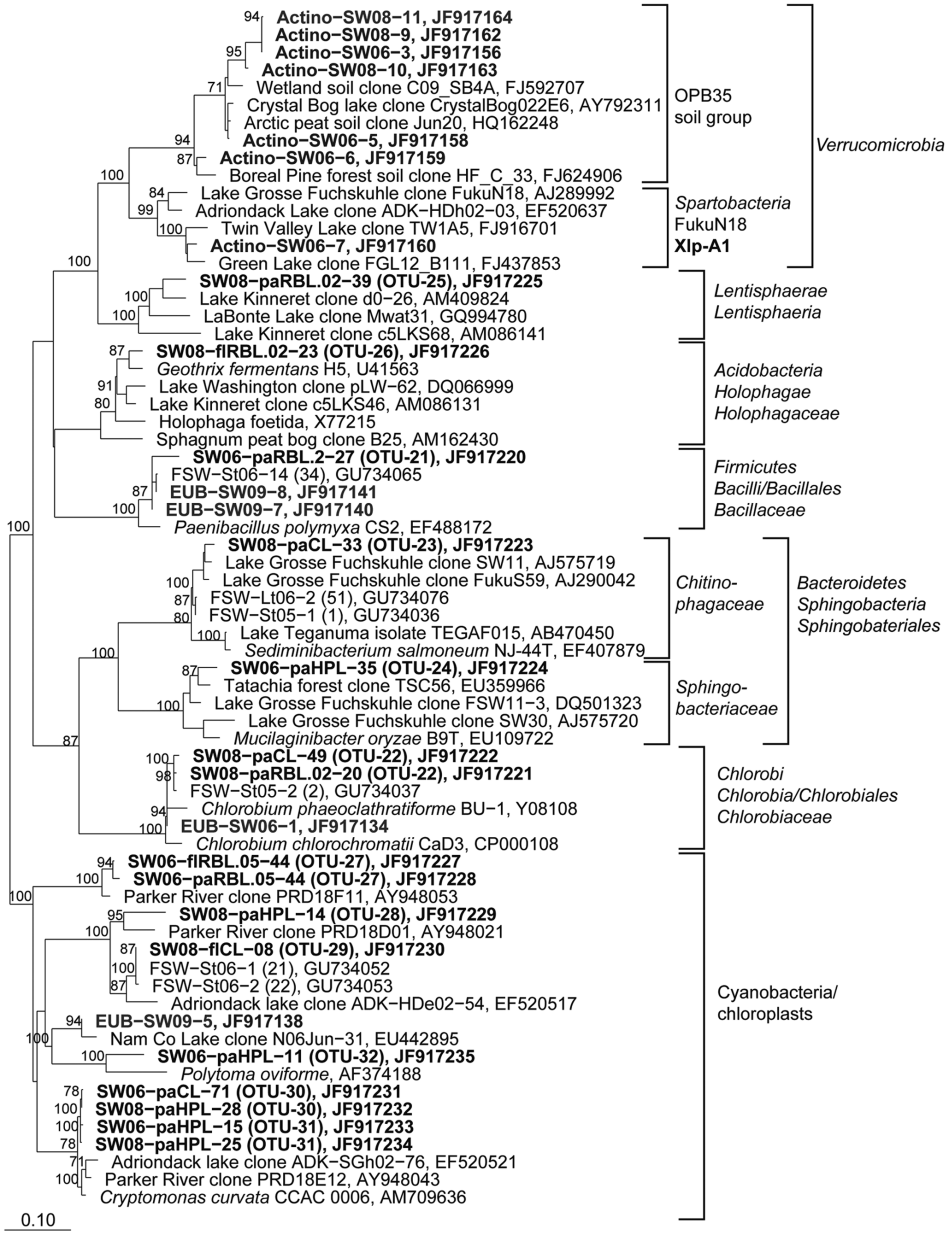
Phylogenetic affiliation of 16S rRNA gene sequences representing OTUs and DGGE bands to the less abundant bacterial groups and chloroplast sequences. Maximum likelihood trees showing the phylogenetic affiliation of OTU and DGGE band sequences to less abundant bacterial groups and chloroplast sequences. Details and abbreviations are indicated in the legend to Figure 5.

Sequencing of clones representing the most abundant operational taxonomic units (OTUs) revealed those bacterial phylotypes causing major changes in BCC upon ROS exposure (Fig. 5–9, Table 2). In 2008, *Polynucleobacter necessarius* (PnecC sub-cluster) represented the most abundant *Betaproteobacteria* phylotype (OTU-1). Increased abundance of *Betaproteobacteria* after ^1^O_2_ exposure was mainly due to *P. necessarius* and a *Limnohabitans*-related phylotype (OTU-3). Both phylotypes decreased after exposure to H_2_O_2_. A second *Polynucleobacter* phylotype (OTU-2) representing the PnecA sub-cluster only occurred in the free-living fractions after H_2_O_2_ addition.

Increased abundance of *Alphaproteobacteria* after ^1^O_2_ exposure was mainly due to OTU-8 representing *Novosphingobium acidiphilum* (Table 2). In addition, increase of an uncultured phylotype (OTU-9) resulted in a highly increased *Alphaproteobacteria* abundance after ^1^O_2_ exposure in 2006. After H_2_O_2_ exposure, in the attached fraction, a *Caulobacteraceae*-related phylotype (OTU-11) increased in relative abundance in 2008 and two other *Alphaproteobacteria* phylotypes (OTU-13/14) in 2006 (Table 2).

Five different phylotypes were responsible for the increased abundance of *Gammaproteobacteria* after high ^1^O_2_ exposure in 2006 (OTU-15 to 19, Table 2). In contrast, only one freshwater-cluster AcI-B phylotype (OTU-20) was responsible for the high abundance of *Actinobacteria* in controls and after H_2_O_2_ exposure.

### Changes in the Composition of Metabolically Active Bacteria

**Figure 10:**
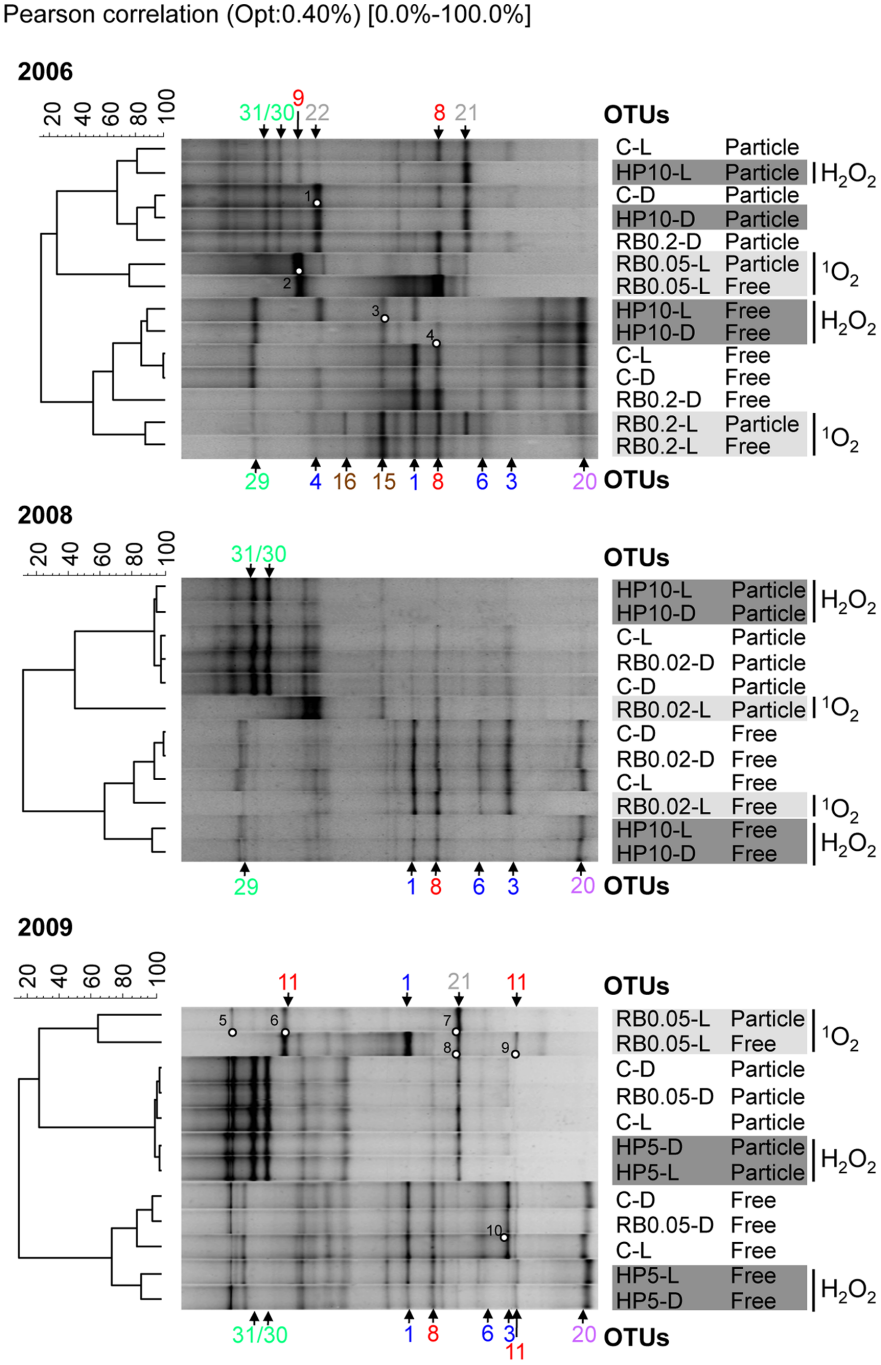
Cluster analysis of *Bacteria* RT-PCR DGGE patterns. Cluster analysis and RT-PCR DGGE patterns of metabolically active free-living (0.22–8 μm in 2006 and 0.22–5 μm in 2008 and 2009) and particle-attached (>8 or >5 μm, respectively) *Bacteria* of *in situ* experiments 2006, 2008 and 2009. Universal *Bacteria* 16S rRNA gene targeting primers were used for analysis. Cluster analyses were performed in GelCompare II version 4.5 (Applied Maths) using unweighted pair-group method using arithmetic average (UPGMA) clustering based on the Pearson correlation which considers the intensity of DGGE bands. Distance matrices are shown in Fig. S4. DGGE bands marked with circles were sequenced. OTU numbers depicted next to the DGGE patterns point at DNA bands identical in DNA sequence (see Table 2). Colours of OTU numbers indicate the phylogenetic affiliation: *Actinobacteria* (purple), *Gammaproteobacteria* (brown), *Alphaproteobacteria* (red), and *Betaproteobacteria* (blue), cyanobacteria/chloroplasts (green), and other *Bacteria* (grey). Phylogenetic affiliations to sequenced DGGE bands are given in Fig. 5–9 and Table S6. Abbreviations are given in Fig. 1.

Analysis of metabolically active bacteria by unweighted pair-group method using arithmetic average (UPGMA) cluster analysis of *Bacteria* RT-PCR Denaturing Gradient Gel Electrophoresis (DGGE) patterns confirmed BCC changes after ^1^O_2_ and H_2_O_2_ exposure as observed by clone library analysis (Fig. 10 and S4). All *in situ* experiments performed in 2006, 2008 and 2009 were repeated within a few days (Fig. S5 A–C).

In UPGMA combining all experiments stable clusters were formed by patterns affiliated with experiments performed in the respective year (data not shown). Therefore, cluster analysis was performed separately for all three years, in which DGGE patterns of particle-attached and free-living bacteria formed separate clusters (Fig. 10) Within these clusters, control experiments (C-L/D, RB-Ds) and H_2_O_2_ treatments (HP-L/D) clustered with each other. In contrast, ^1^O_2_ exposure caused more pronounced changes in DGGE banding patterns. Particle-attached and free-living fractions in 2006 and 2009 were found in the same cluster after 2.8 and 1.9-fold (RB0.05-L, 2006 and 2009) and after 8-fold (RB0.2-L, 2006) ^1^O_2_ increase. After moderate ^1^O_2_ exposure (RB0.05-Ls), changes in DGGE bands representing the uncultured *Alphaproteobacterium* OTU-9 and the *Firmicutes* OTU-21 in both particle-attached and free-living fractions greatly affected cluster formation. At higher ^1^O_2_ exposure (RB0.2-L), however, DGGE banding patterns of the free-living fraction were similar to the respective controls (Fig. 10) represented by *P. necessarius* OTU-1, *N. acidiphilum* OTU-8, and *Methylococcaceae* OTU-15. In 2008, slightly increased ^1^O_2_ exposure (RB0.02-L) had a minor effect on BCC and the respective DGGE clusters were similar to the controls. In all three experiments, disappearance of the DGGE band representing AcI-B *Actinobacteria* OTU-20 comprised the most obvious change in community composition of free-living bacteria after ^1^O_2_ exposure (Table 2).

BCC changes after H_2_O_2_ exposure were generally caused by i) decreased intensity of DGGE bands representing *P. necessarius* OTU-1 and *N. acidiphilum* OTU-8 and ii) the absence of DGGE bands representing *Limnohabitans-*related OTU-3/6. These changes occurred in different extent in free-living fractions of all three experiments and also partially in the respective particle-attached fractions.

### Phylotype-specific Changes within Major Bacterial Groups

*Betaproteobacteria*, *Actinobacteria*, and *Sphingomonadaceae-*specific RT-PCR DGGE analysis increased the phylogenetic resolution of our study and revealed separate clusters for free-living and particle-attached bacteria by UPGMA analysis (Fig. S6). After ^1^O_2_ exposure (RB-Ls), DGGE banding patterns obtained for all three bacterial groups were separated from controls, whereas after H_2_O_2_ exposure, the DGGE bands always clustered together with controls.

**Figure 11:**
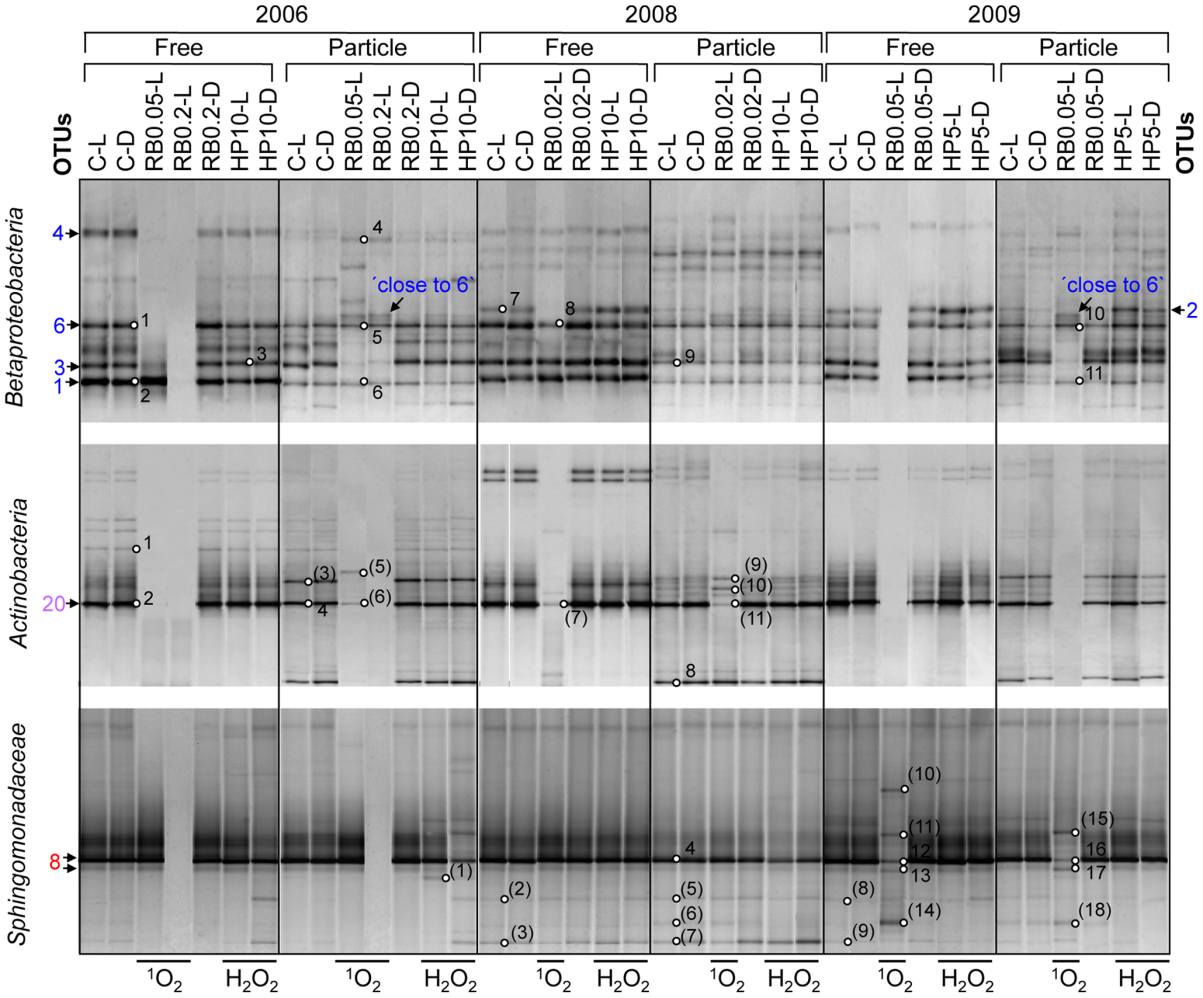
Group specific RT-PCR DGGE analysis. RT-PCR DGGE analysis of metabolically active free-living (0.22–8 μm in 2006 and 0.22–5 μm in 2008 and 2009) and particle-attached (>8 or >5 μm, respectively) *Betaproteobacteria*, *Actinobacteria*, and *Sphingomonadaceae* after ^1^O_2_ and H_2_O_2_ exposure. Group-specific 16S rRNA gene targeting primer-systems were used for analysis. All treatments of *in situ* experiments 2006, 2008 and 2009 were investigated. DGGE bands marked with circles were sequenced. DGGE band numbers in brackets were not affiliated to the investigated groups. Numbers with arrows show the assignment to respective OTUs (see Table 2). Abbreviations are given in Fig. 1.

Major DGGE bands of both *Betaproteobacteria* fractions represented *P. necessarius* OTU-1 and *Limnohabitans-*related OTU-3/6 (Fig. 11). In 2008 and 2009, the DGGE band representing PnecA OTU-2 was observed with higher intensities in the free-living fractions. Singlet oxygen exposure resulted in different effects on phylotype composition of free-living vs. particle-attached *Betaproteobacteria*. The 2.8-fold increased ^1^O_2_ exposure decreased diversity of free-living *Betaproteobacteria* to solely 2 DGGE bands in 2006 represented by *P. necessarius* OTU-1 and *Limnohabitans-*related OTU-3. The 8-fold increased ^1^O_2_ exposure diminished all free-living *Betaproteobacteria*, whereas DGGE bands of particle-attached *Betaproteobacteria* representing *P. necessarius* OTU-1 and *Limnohabitans-*related OTU-6 were not affected by ^1^O_2_ exposure. In the same treatment, an additional DGGE band representing a phylotype closely related to OTU-6 occurred (DGGE band 5, Fig. 11). In 2008, the much lower ^1^O_2_ exposure led to the disappearance of a DGGE band in the free-living fraction representing PnecA OTU-2. The same DGGE band became more intense after H_2_O_2_ exposure in both, particle-attached and free-living fractions of 2008 and 2009. In general, the effects of ^1^O_2_ exposure on *Betaproteobacteria* in 2006 were confirmed in 2009 whereby the 1.9-fold increased ^1^O_2_ exposure in 2009 had similar effects compared to the 8-fold increased ^1^O_2_ exposure in 2006.

The AcI-B OTU-20 represented the most abundant *Actinobacteria* DGGE band of free-living and particle-attached fractions. However, the relative abundance of *Actinobacteria* was low on particles as revealed by clone-library (Fig. 4) and *Bacteria* RT-PCR DGGE analysis (Fig. 10). After ^1^O_2_ exposure, *Actinobacteria* DGGE bands were lacking, except in 2008 when a DGGE band representing a *Mycobacteria-*related phylotype occurred (band 8, Fig. 8 and 11). Other DGGE bands present after ^1^O_2_ exposure belonged to the *Verrucomicrobia* (Fig. 9 and 11).

*Sphingomonadaceae*-specific RT-PCR DGGE analysis indicated that *N. acidiphilum* (OTU-8) was the pre-dominant *Sphingomonadaceae* in the SW basin. Only high ^1^O_2_ exposure affected the intensity of its respective DGGE band (Fig. 11).

## Discussion

### Comparison of ^1^O_2_ and H_2_O_2_ Toxicity

Moderately increased ^1^O_2_ and highly increased H_2_O_2_ concentrations caused similar inhibition of ^14^C-leucine incorporation suggesting different toxic potentials of ^1^O_2_ and H_2_O_2_. This finding also indicates that small changes of ^1^O_2_ generation (frequent during diurnal changes in sunlight intensity) may hamper microbial activity in surface waters of humic lakes. In contrast, only large changes in H_2_O_2_ concentrations may affect the activity of dominant bacterial species. However, the H_2_O_2_ concentrations applied in our experiments were not exaggerated and the natural potential of H_2_O_2_ formation in 0.22 μm filtered lake water of the SW basin was high (Fig. S7). In H_2_O_2_ depleted water samples, H_2_O_2_ concentrations in the μM range can be reached rapidly after irradiation with sunlight or UV-A/B which has been frequently observed for boreal lakes [25], [26]. Microorganisms strongly contribute to the decay of H_2_O_2_ [27]. This is indicated by 2.4-fold higher H_2_O_2_ decay rates in our unfiltered water samples compared to those filtered through 0.22 μm (Materials S1). Obviously, the bacterial community or at least some phylotypes can detoxify H_2_O_2_ and therefore balances H_2_O_2_ levels in their environment. This notion is in line with earlier findings that bacteria are involved in H_2_O_2_ degradation in marine surface waters [27] and that H_2_O_2_ degradation by some bacterial populations is important for growth of other bacteria in aquatic environments [28]. Hence, bacteria thriving in surface waters of humic lakes are well adapted to H_2_O_2_ exposure and may prevent accumulation of toxic H_2_O_2_ concentrations.

### Contrasting Effects of ^1^O_2_ and H_2_O_2_ on *Actinobacteria* and *Betaproteobacteria*

AcI-B *Actinobacteria* and betII lineage *Betaproteobacteria* mainly of the PnecC sub-cluster are the most abundant bacterial groups in the SW basin [29], [30], [31]. *Actinobacteria* of the AcI-B cluster are low in abundance on particles [32]. Their high sensitivity to ^1^O_2_ and the finding that humic matter rich particles generate high amounts of ^1^O_2_ [23] could explain the obvious absence of AcI-B *Actinobacteria* from particles. Contrary, AcI-B *Actinobacteria* of the SW basin were more resistant to H_2_O_2_ exposure. Thus, it is likely that AcI-B *Actinobacteria* produce peroxidases to degrade recalcitrant organic matter and contribute to the high overall extracellular peroxidase activity in Lake Grosse Fuchskuhle [33]. This life-style requires increased resistance to peroxides and thus may explain the high relative abundance of AcI-B *Actinobacteria* at increased H_2_O_2_ concentrations. A recently analysed single cell genome of the AcI-B lineage supports this notion, because several genes encoding glutathione depended peroxiredoxins were identified that potentially account for the proposed resistance against peroxides [34].

In Lake Grosse Fuchskuhle and in other freshwater ecosystems the abundances of *Actinobacteria* and *Betaproteobacteria* are negatively correlated [14], [30], and *Actinobacteria* numbers are usually lower in summer months. The addition of photo-chemically modified DOM to water samples increased *Actinobacteria* abundance [14]. By irradiating DOM high amounts of H_2_O_2_ accumulate [11], and the subsequent incubation in the dark excludes formation of ^1^O_2_. Therefore, only effects of H_2_O_2_ on bacterial dynamics can be monitored by such assays. *Actinobacteria* had a high resistance against H_2_O_2_ in our study. In contrast, several *Betaproteobacteria* phylotypes detected in our study were H_2_O_2_ sensitive, but resistant to ^1^O_2_ exposure. Consequently, the negative correlation between *Actinobacteria* and *Betaproteobacteria* in the SW basin is at least partly the result of their contrasting resistance and sensitivity to ^1^O_2_ and H_2_O_2_.

High solar radiation causes high ^1^O_2_ exposure during the summer months and may result in reduced AcI-B *Actinobacteria* abundance. In contrast, *P. necessarius* was favoured by increasing ^1^O_2_ concentrations and generally shows highest abundance and activities in summer [35] and it also grows well on photodegradation products of humic matter, such as acetate [36], [37], [38]. AcI-B *Actinobacteria* are more abundant in autumn and early spring [30], [32] when input of unbleached NOM from the adjacent fen into the SW basin is high. This unbleached NOM generates much more H_2_O_2_ than ^1^O_2_ (Materials S1) and may be a key regulator of the observed opposing dynamics of AcI-B *Actinobacteria* vs. *Betaproteobacteria*.

### *Alpha-* and *Gammaproteobacteria* Resist High ^1^O_2_ Doses

*Alpha-* and *Gammaproteobacteria* are two major lineages of freshwater bacteria, which have gained relatively little attention in the past [39]. Our data and previously published clone libraries [29], [30], [31] indicate the persistence of *N. acidiphilum* in the humic matter rich SW basin. Its relative abundance strongly increased during ^1^O_2_ exposure suggesting a high ^1^O_2_ resistance which can be explained by a high cellular carotenoid content [40]. In addition, *Sphingomonadaceae* are known to degrade aromatic compounds and *N. acidiphilum* was favoured by the addition of phenol that represents an important fraction of leached DOM [41]. Thus, cellular quenching of ^1^O_2_ by carotenoids and the use of aromatic compounds are features of *N. acidiphilum*, which may well explain its persistence in humic matter rich systems.

The increase in relative abundance of several *Alpha*- and *Gammaproteobacteria* after ^1^O_2_ exposure may be related to specific defence-systems protecting, for example, anoxygenic phototrophic *Alphaproteobacteria* against ^1^O_2_ damages [42], [43]. This is supported by the recent finding that anoxygenic phototrophic bacteria of the SW-basin mainly consist of *Alphaproteobacteria* [44]. The key regulators controlling such cellular responses include specific RNA polymerase sigma factors and have been found in the genomes of several *Alpha*- and *Gammaproteobacteria* lineages [45] including non-phototrophic *Caulobacter crescentus* [46]. Thus, induction of ^1^O_2_-specific defence-systems may explain the increased relative abundance of the *Caulobacteraceae*-related phylotype (OTU-11) in the present study.

### Particle-attached Phylotypes are More Resistant to ^1^O_2_ Exposure

Particles represent hotspots of bacterial activity in aquatic environments [47]. Humic matter rich particles have been shown to generate higher ^1^O_2_ concentrations compared to the surrounding water by the application of hydrophobic ^1^O_2_ traps [23]. Recent studies could not verify a high ^1^O_2_ generation in humic particles [48] or suggest that ^1^O_2_ is quenched by certain reactive groups [49]. Our study revealed the existence of particle-associated phylotypes that were obviously more resistant to ^1^O_2_ exposure than their free-living counterparts. Particle-attached bacteria represented by *P. necessarius* OTU-1 and the *Limnohabitans*-related OTU-6 were indeed more resistant to ^1^O_2_ exposure than their free-living counterparts. Particle-associated bacteria exhibit different lifestyles and thus often represent different ecotypes [50], which requires also adaptation to different levels of oxidative stress. Alternatively, phylotypes in particle-attached and free-living fractions may represent the same ecotypes, whereby inducible response mechanisms against increased oxidative stress should allow for colonization of particles in the upper, well-illuminated water layers. Furthermore, it cannot be fully excluded that *P. necessarius* 16S rRNA gene sequences in the particle-attached fraction (>5 μm) originate from ciliate endosymbionts, namely *Stentor amesthystinus* (Dziallas and Grossart, unpubl. data). In contrast, highly ^1^O_2_ sensitive AcI-B *Actinobacteria* were absent from humic particles representing nutrient, but ^1^O_2_ rich microhabitats (see above).

### Defence Mechanisms Against Environmental ROS Exposure

Details on the presence of molecular response mechanisms against environmental ROS exposure in typical freshwater bacteria are elusive. Recently, molecular defence systems against ^1^O_2_ exposure were found in bacteria [42], [43] and defence strategies against H_2_O_2_ generated in aerobic metabolism are known in detail for several bacterial model systems [21].

Carotenoids are inevitable in photosynthetic bacteria and in the chloroplasts of plants to prevent photosystem based generation of ^1^O_2_ [42], [43]. Non-photosynthetic bacteria also exhibit carotenoids, which likely serve as quenchers of ^1^O_2_ generated by cellular photosensitizers such as flavins [42] or by various extracellular sources. Cellular scavengers, which include amino acids such as L-histidine and trypotphan, reduced thiols (glutathione, thioredoxin), mycosoprine lysine and polyamines also minimize cellular damages by ^1^O_2_. Such scavengers need to be regenerated after their reaction with ^1^O_2_, and therefore enzymes involved in adjusting the cellular redox homeostasis need to be activated (reviewed in [43]).

In photosynthetic *Alphaproteobacteria*, response mechanisms to ^1^O_2_ exposure are controlled by the alternative sigmafactor RpoE, which is bound to the anti-sigmafactor ChrR under non-stress conditions. The release of RpoE from ChrR after ^1^O_2_ exposure triggers the induction of genes encoding stress response mechanisms and further regulatory factors, including RpoH_II_ and several small regulatory RNAs [42]. Homologs of these sigmafactors are conserved in photosynthetic *Alphaproteobacteria* and have been found in several *Beta-* and *Gammaproteobacteria* lineages [45]. Genomes of species representing abundant freshwater bacterial clades did not harbour homologous genes. Hence, defence systems and their control in abundant freshwater bacteria may substantially differ from established bacterial model systems.

Very likely, individual bacterial lineages use different strategies to overcome natural ^1^O_2_ exposure, which could explain very well the species specific sensitivity to ^1^O_2_ exposure in our study.

Hydrogen peroxide is detoxified by cellular enzymes such as catalases and peroxidases (glutathione peroxidases and peroxiredoxin) [21]. Increased H_2_O_2_ concentrations lead to rapid cell death by the oxidation and disassembly of iron-sulphur clusters, which are common in electron transport chain components. Hydrogen peroxide together with free iron(II) leads to the formation of highly toxic hydroxyl radials by the Fenton reaction, which rapidly react with most cellular components and facilitate cell mortality. Therefore, cellular levels of H_2_O_2_ are tightly balanced and the cellular response is well regulated by, for example, OxyR or PerR which coordinate genes for H_2_O_2_ degradation, glutathione turnover, production of redox buffers as glutaredoxin and thioredoxin as well as genes involved in controlling iron metabolism. All bacteria with an aerobic metabolism, therefore, require defence systems against H_2_O_2_ exposure. This may explain, why H_2_O_2_ had a much smaller effect on BCC compared to ^1^O_2_ in the environment.

### Niche Separation of Closely Related Species Caused by Exposure to Different ROS

Our experiments in 2008 indicate niche separation of closely related *Polynucleobacter* phylotypes by moderately increased ^1^O_2_ exposure. The *Polynucleobacter* phylotype of the PnecC sub-cluster (OTU-1) was highly resistant against exposure to ^1^O_2_, but negatively affected by H_2_O_2_. In contrast, the *Polynucleobacter* phylotype of the PnecA sub-cluster (OTU-2) was only detected after H_2_O_2_ exposure in clone libraries of free-living bacteria. Additionally, a corresponding DGGE band was observed in all free-living fractions by *Betaproteobacteria-*specific RT-PCR DGGE analysis, except after intense ^1^O_2_ exposure. Hence, ecological niches of those related phylotypes might be separated by variations in their sensitivity to ^1^O_2_ and H_2_O_2_. In line with our finding, occurrence of the *Polynucleobacter* sub-cluster PnecA and PnecC depends on lake colour [51], most likely because H_2_O_2_ formation largely depends on concentration and quality of NOM [25]. Moreover, the presence of various *Polynucleobacter* sub-clusters may also reflect the availability of different substrates since *Polynucleobacter* species assimilate low-molecular-weight substances [38] that can be also generated by photochemical NOM degradation.

We further observed ROS dependent niche separation for *Limnohabitans*-related phylotypes. *Betaproteobacteria*-specific RT-PCR DGGE patterns revealed the occurrence of a *Limnohabitans*-related phylotype closely related to OTU-6′ on particles after increasing ^1^O_2_ exposure in 2006 and 2009. This phylotype was also enriched after long-term exposure with moderately increased ^1^O_2_, whereas the OTU-6 phylotype only occurred in the respective controls [16] indicating a lower ^1^O_2_ resistance. Fortunately, we were able to isolate a respective strain from the SW basin and found an efficient adaptation to inhibitory ^1^O_2_ exposure by pre-incubation with non-inhibitory ^1^O_2_ concentrations (data not shown). This notion suggests that highly effective response mechanisms to increased ^1^O_2_ may be present in this specific *Limnohabitans* strain. Niche separation of coexisting closely related *Limnohabitans* strains has been shown recently [52], but in this case it was caused by differences in predation and virus infections. Niche separation of closely related phylotypes of *Limnohabitans* by ^1^O_2_ exposure underlines our hypothesis that different ROS affect BCC in a highly phylotype-specific manner, particularly in humic matter rich lakes.

## Conclusions

From our data we conclude that differences in sensitivity to ^1^O_2_ and H_2_O_2_ may explain the negative correlation in abundance of *Actinobacteria* and *Betaproteobacteria* in the surface waters of Lake Grosse Fuchskuhle and elsewhere. The exclusion of specific bacterial lineages from humic matter rich particles and the presence of species-like taxa due to ROS specific separation of ecological niches should be regarded as an ecological factor shaping natural microbial communities. Hence, temporal and spatial differences in ROS generation, particularly in humic matter rich aquatic ecosystems, have the potential to affect major microbial processes and their rates. For example, niche separation by ROS has strong implications for bacterial adaptation and evolution in natural ecosystems. We propose that changes in ^1^O_2_ exposure have a larger impact on BCC than H_2_O_2_, because ^1^O_2_ is i) more toxic compared to H_2_O_2_ and ii) defence mechanisms against H_2_O_2_ are present in all aerobic organisms, whereas putative defences against singlet oxygen exposure may only occur in bacteria specifically adapted to cellular or environmental ^1^O_2_ formation. Further, insights into the molecular mechanisms of cellular defences against environmental ROS in general and singlet oxygen in particular are necessary to understand in detail the role of ^1^O_2_ and H_2_O_2_ for controlling activity and composition of aquatic microbial communities.

## Materials and Methods

### Study Site

All field studies were conducted in the humic acid rich south-west basin of the artificially divided dystrophic Lake Grosse Fuchskuhle [52]. Physico-chemical parameters of the lake were described previously [16], [29], [30] and are compiled for all experimental periods in Table S3.

The IGB is authorized by the Landkreis Oberhavel to obtain samples from Lake Grosse Fuchskuhle and to conduct mesocosm experiments as performed in our study. Our studies did not endanger protected wildlife in or around the lake.

### Sampling and Experimental Conditions

Subsurface water samples were collected in autoclaved Pyrex-glass bottles on the same day prior to the start of *in situ* exposure experiments. All set ups were prepared in a dark shelter at the lake shore and water samples were subsequently incubated 10 cm below the water surface in the humic SW basin of Lake Grosse Fuchskuhle.

Generation of ^1^O_2_ was artificially increased by adding 0.02 to 0.2 μM of the photosensitizer Rose Bengal under sunlight exposure (RB-L). Concentrations of H_2_O_2_ were experimentally increased by adding 5–10 μM H_2_O_2_ to enhance peroxide stress in light and dark incubations (HP-L and HP-D). Controls included light and dark incubations of natural lake water (C-L and C–D) without addition of any chemicals and a RB dark control (RB-D).

For the first experiment on 12^th^ July 2006 [16], 1 L water samples were incubated in polypropylene bags (Carl Roth, Karlsruhe, Germany) between 13∶30 and 18∶00. The light treatments were repeated in 2006 on 14^th^ July (C-L), 15^th^ July (RB0.2-L), 18^th^ July (RB0.05-L) and 20^th^ July (HP-L). In each experiment we compared the exposure to the untreated control obtained at the start of the experiment. The second experiment was performed on 5^th^ September 2008 by incubating 400 mL water samples in polyethylene Whirl-Pak Bags (Nasco, Fort Atkinson, WI, USA) between 12∶15 and 16∶15. Prior to incubations, water samples were diluted with an equal volume of 0.22 μm pre-filtered surface water. A replicate of this experiment was performed on 4^th^ September. In the third experiment on 14^th^ August 2009, we incubated 500 mL water samples in Whirl-Pak Bags between 9∶00 and 13∶00. All Whirl-Pak bags were covered with UV-A/B absorbing polyester sheets 90 NR (Modulor, Berlin) to exclude effects of UV-A/B radiation. The experiment was repeated in triplicates on 17^th^ August. Transmission spectra are given in Fig. S8 for plastic bags and sheets, respectively.

Solar radiation and rainfall affects the NOM reactivity in the lake. In order to monitor pre-experiment weather conditions, weather data for 30 day prior to the each experiments were obtained from the weather station in Menz (53°10′ N, 13°05′ E). Menz is closely located to Lake Grosse Fuchskuhle. The data were purchased from the Deutscher Wetterdienst (www.dwd.de) and depicted in the Figure S9.

### Measurement of ^1^O_2_ and H_2_O_2_

ROS concentrations were determined in 0.22 μm filtered water samples. Singlet oxygen steady state concentrations ([^1^O_2_]_SS_) were measured using furfuryl alcohol [24] as described previously [16]. Concentrations of H_2_O_2_ were measured by using the Amplex Red method [53] with slight modifications (Materials S1). Analysis were performed in triplicates. Differences between treatments were analysed by one-way ANOVA followed by pair-wise multiple comparison analysis with the Tukey test (Sigma Stat version 2.0, Systat Software, Richmond, California, USA).

### Bacterial Numbers and Microbial Activity

Bacteria cell numbers were determined by Sybr Green I staining and epifluorescence microscopy [16]. Microbial activity was measured by [^14^C]-leucine incorporation [54]. Sample-triplicates (5 mL) and formalin-fixed controls were incubated immediately after experiments with [^14^C]-leucine (1.15×10^10^ Bq mmol^−1^; Amersham) for 1 h at *in situ* temperature in the dark. Incubations were stopped by formalin addition.

### Simultaneous DNA and RNA Extraction from Water Samples

All *in situ* experiments performed in 2006, 2008 and 2009 were repeated within a few days. Water samples were immediately put on ice prior to filtration. Particle-attached bacteria were collected on 8 μm cellulose-nitrate membranes (Satorius, Göttingen, Germany) in 2006 or on 5 μm sterile Minisart syringe filters (Sartorius) in 2008 and 2009. Free-living bacteria from the 5 μm filtrates were collected on 0.22 μm Sterivex™-GP filter units (Millipore, Schwalbach, Germany) and filters were immediately stored at −80°C. Triplicates from the second experiment performed in 2009 were pooled prior to the extraction of nucleic acids. DNA and RNA were extracted simultaneously as described by [55]. Reaction volumes were decreased for the use of 2-ml tubes. Precipitated nucleic acids were resuspended in 100 μl RNase/DNase-free water (Carl Roth). RNA extracts were treated with 1 U RQ1 DNase (Promega, Madison, WI, USA) and purified with phenol/chloroform (2006) or were incubated with 1 U DNase I (Fermentas, St. Leon-Rot, Germany), which was subsequently heat-inactivated (2008 and 2009).

### 16S rRNA Gene Clone Libraries and RT-PCR DGGE

Bacterial 16S rRNA gene clone libraries were generated with primers 8F and 1492R [56] and operational taxonomic units (OTUs) were defined by Amplified Ribosomal DNA Restriction Analysis (ARDRA) [57]. Community changes of metabolically active *Bacteria, Actinobacteria, Betaproteobacteria*, and *Sphingomonadaceae* were investigated by 16S rRNA targeting RT-PCR DGGE. Details are given in Materials S1 and Tables S4 and S5.

### Phylogenetic Analysis of 16S rRNA Gene Sequences

Sequences were aligned with the SINA Web aligner (http://www.arb-silva.de/aligner/) and analysed in ARB [58] using the SILVA SSURef NR 104 database [59]. Maximum likelihood trees were constructed with using RAxML v7.04 [60] with GTR-GAMMA and rapid bootstrap analysis. Trees were generated with nearly full-length sequences (>1300 nt) spanning *E. coli* positions 56 to 1444 [61]. Tree topologies were confirmed by the generation of trees using *Proteobacteria*, *Actinobacteria*, and *Bacteria* 50% base frequency filters. Partial sequences were added with ARB parsimony without changing the overall tree topology. Sequences are deposited in GenBank with accession numbers JF917134–JF917235, JF925281, and JF925282.

## Supporting Information

Figure S1 **Activity of heterotrophic bacteria after ^1^O_2_ and H_2_O_2_ exposure.**
(PDF)

Figure S2 **Cell numbers in controls and in ^1^O_2_ and H_2_O_2_ treatments.**
(PDF)

Figure S3 **Rarefaction analysis of nearly full-length 16S rRNA gene clone libraries.**
(PDF)

Figure S4 **Distance matrices of Pearson correlation based UPGMA cluster analysis.**
(PDF)

Figure S5 **Robustness of changes in BCC observed by RT-PCR-DGGE analysis shown by repeats of the 2006 (A), 2008 (B) and 2009 (C)** ***in situ*** **experiments.**
(PDF)

Figure S6 **Cluster analysis of** ***Betaproteobacteria, Actinobacteria,*** **and** ***Sphingomonadaceae-*** **specific 16S rRNA gene based RT-PCR DGGE analysis.**
(PDF)

Figure S7 **Delayed formation of hydrogen peroxide (H_2_O_2_) in 0.22 μm filtered water samples exposed to natural sunlight.**
(PDF)

Figure S8 **Transmission scans of poly-propylene (PP) bags, poly-ethylene (PE) Whirl-Pak bags, and the UVA/B block sheet.**
(PDF)

Figure S9 **Weather data for 30 day prior to the experiments carried out in 2006, 2008, and 2009.**
(PDF)

Table S1 **NOM concentrations and reactivity of surface water samples from the SW basin of Lake Grosse Fuchskuhle.**
(PDF)

Table S2 **Cell numbers of** ***in situ*** **incubation experiments in 2006 and 2008.**
(PDF)

Table S3 **Physico-chemical parameters of Lake Grosse Fuchskuhle SW compartment.**
(PDF)

Table S4 **Sequences of 16S rRNA gene targeting oligonucleotide primer used for RT-PCR DGGE analysis.**
(PDF)

Table S5 **PCR and RT-PCR programs for the amplification of 16S rRNA and 16S rRNA gene fragments of** ***Bacteria*** **and bacterial subgroups used for DGGE fingerprint analysis.**
(PDF)

Table S6 **Phylogenetic affiliation of 16S rRNA gene sequences representing DGGE bands.**
(PDF)

Materials S1 **Characterization of water sample photo-reactivity, including NOM characteristics of south-west compartment samples, comparison of water sample photo-reactivity, in situ H_2_O_2_ formation and decay, and the potential photochemical effects of unbleached material from the acidic fen area.** Investigation of bacterial community composition by 16S rRNA (gene) based methods by the generation and screening of 16S rRNA gene clone libraries, 16S rRNA targeting reverse transcriptase (RT)-PCR DGGE analysis and group-specific 16S rRNA targeting RT-PCR DGGE analysis.
(PDF)
