# Supplementary material for: Contrasting Effects of Singlet Oxygen and Hydrogen Peroxide on Bacterial Community Composition in a Humic Lake
Source: PLoS One. 2014 Mar 25;9(3):e92518. doi: 10.1371/journal.pone.0092518 (PMC3965437; doi:10.1371/journal.pone.0092518)
Supplement: Figure S1 — Activity of heterotrophic bacteria after 1O2 and H2O2 exposure. (PDF) [file pone.0092518.s001.pdf]

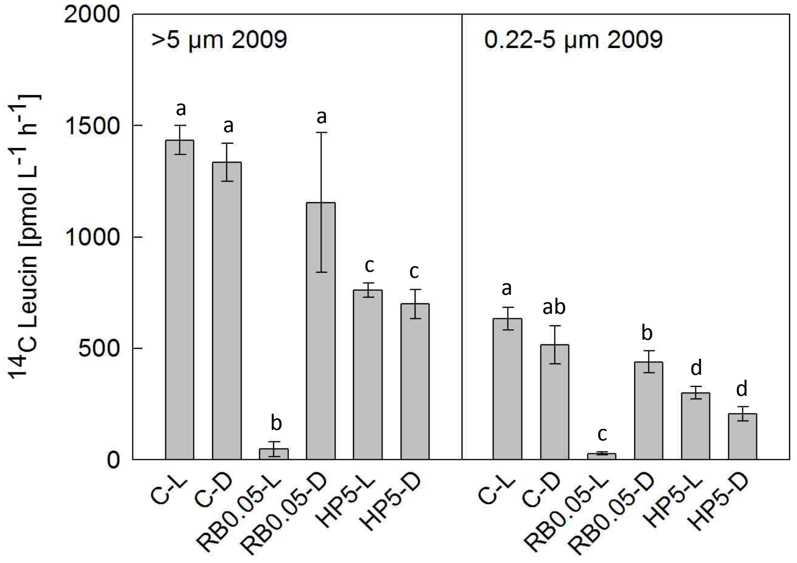

Figure S1

Activity of heterotrophic bacteria after $^1O_2$ and H_2O_2 exposure – separate analysis of particle-attached (>5 µm) and free-living (5 – 0.22 µm) fractions in experiments of 14th August 2009. Activity of heterotrophic bacteria was determined by ^{14}C-labeled leucine incorporation. Different letters at top of the bars depict significant differences between values as determined by one-way ANOVA followed by pair-wise multiple comparison analysis with the Tukey test. C-L/D: Light and dark control incubations, RB-L: Light incubation with increased $[^1O_2]_{SS}$, RB-D: Dark control for RB, HP-L/D: Light and dark incubations with H_2O_2. Numbers behind RB and HP represent µM concentrations used of RB and H_2O_2.
