# Supplementary material for: Contrasting Effects of Singlet Oxygen and Hydrogen Peroxide on Bacterial Community Composition in a Humic Lake
Source: PLoS One. 2014 Mar 25;9(3):e92518. doi: 10.1371/journal.pone.0092518 (PMC3965437; doi:10.1371/journal.pone.0092518)
Supplement: Figure S2 — Cell numbers in controls and in 1O2 and H2O2 treatments. (PDF) [file pone.0092518.s002.pdf]

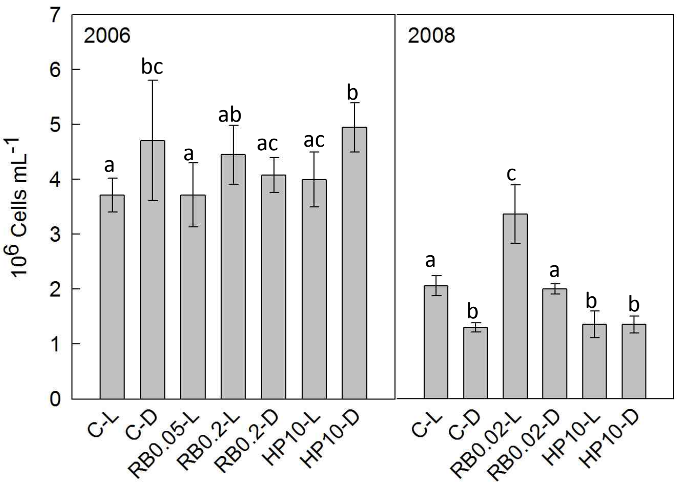


**Figure S2**

Cell numbers in controls and in $^1O_2$ and $H_2O_2$ treatments. Total cell counts of Sybr Green I stained cells filtered onto 0.22 µm membrane filters were determined for experiments in 2006 and 2008. Error bars indicate standard deviations of ten counts. Different letters at top of the bars depict significant differences between values as determined by one-way ANOVA followed by pair-wise multiple comparison analysis with the Tukey's test. Tests were done separately for each year. Abbreviations are given in Figure S3.
