# Supplementary material for: Contrasting Effects of Singlet Oxygen and Hydrogen Peroxide on Bacterial Community Composition in a Humic Lake
Source: PLoS One. 2014 Mar 25;9(3):e92518. doi: 10.1371/journal.pone.0092518 (PMC3965437; doi:10.1371/journal.pone.0092518)
Supplement: Figure S3 — Rarefaction analysis of nearly full-length 16S rRNA gene clone libraries. (PDF) [file pone.0092518.s003.pdf]

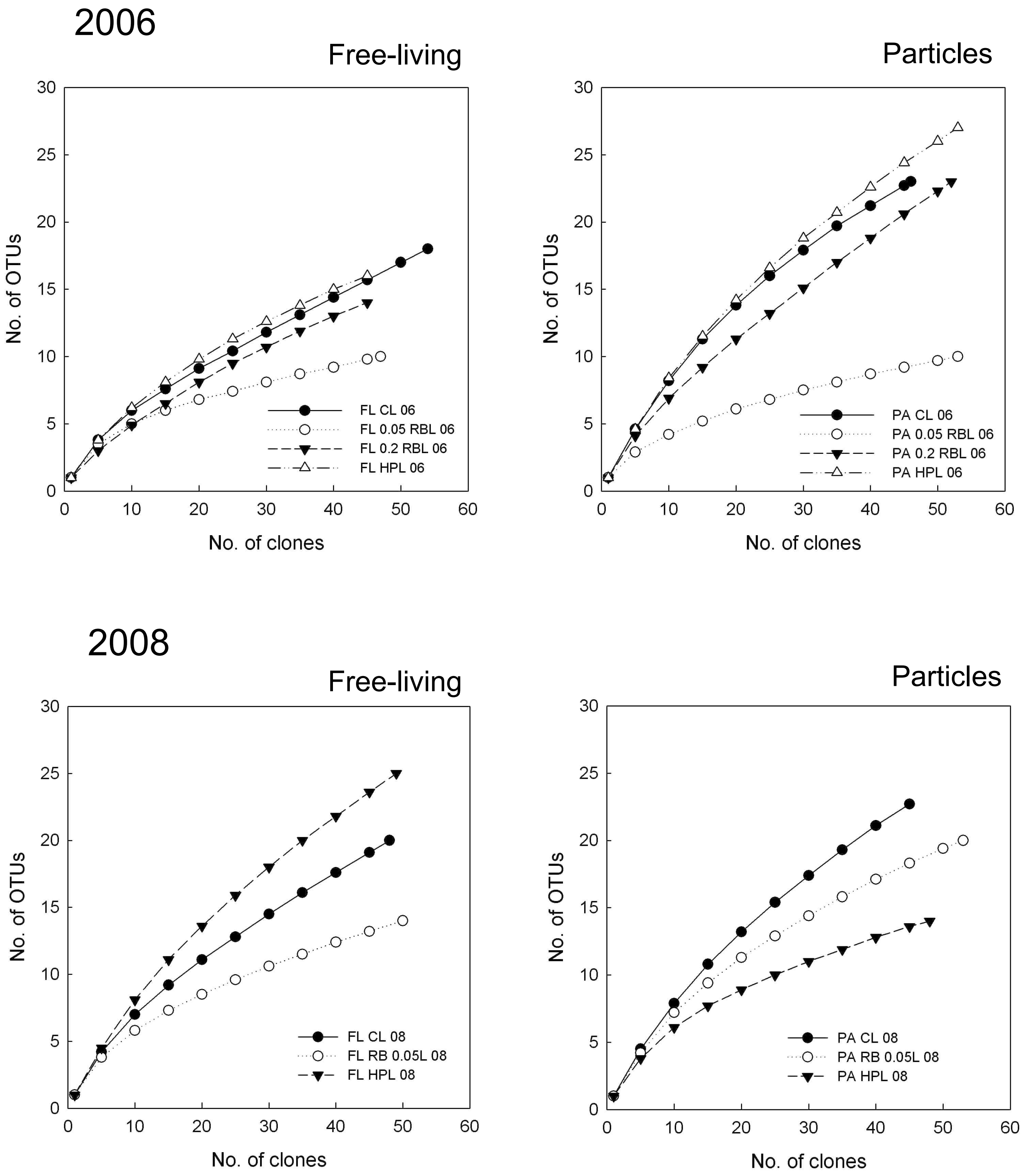

Figure S3

Rarefaction analysis of nearly full-length 16S rRNA gene clone libraries generated for the treatments with increased $^{1}O_2$ (RB-L), H_2O_2 light incubations (HP-L), and light controls (C-L) 2006 and 2008, respectively. Free-living (0.22-8 µm in 2006 and 0.22-5 µm in 2008 and 2009, FL) and particle-attached (>8 or >5 µm, respectively, PA) bacterioplankton were investigated separately, respectively. Approximately 50 clones were investigated for each clone library. OTUs were defined from combined ARDRA pattern generated with HaeIII and RsaI.
