# Supplementary material for: Contrasting Effects of Singlet Oxygen and Hydrogen Peroxide on Bacterial Community Composition in a Humic Lake
Source: PLoS One. 2014 Mar 25;9(3):e92518. doi: 10.1371/journal.pone.0092518 (PMC3965437; doi:10.1371/journal.pone.0092518)
Supplement: Figure S4 — Distance matrices of Pearson correlation based UPGMA cluster analysis. (PDF) [file pone.0092518.s004.pdf]

Pearson correlation (Opt:0.40%) [0.0%-100.0%]

2006

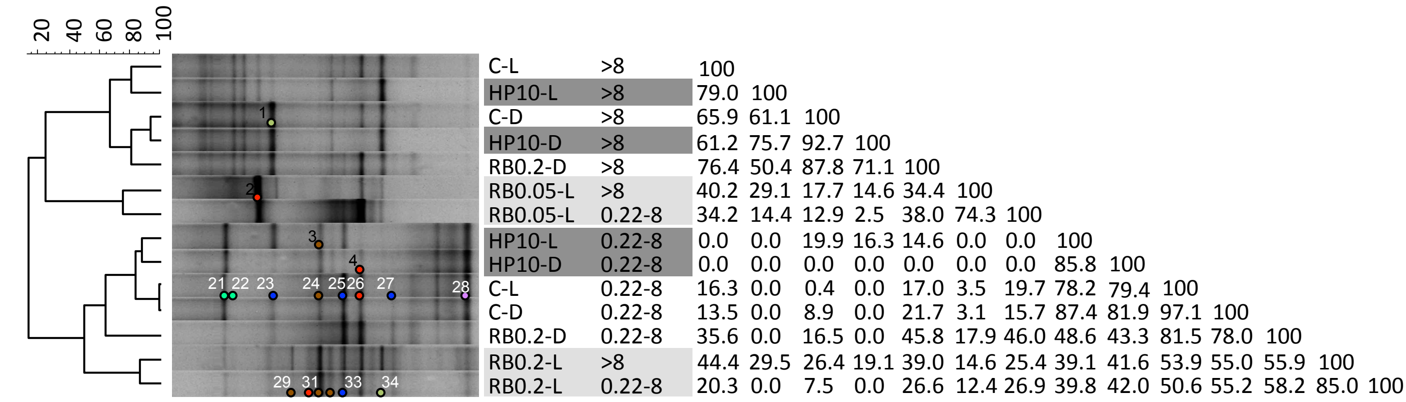

C-L	>8	100													
HP10-L	>8	79.0	100												
C-D	>8	65.9	61.1	100											
HP10-D	>8	61.2	75.7	92.7	100										
RB0.2-D	>8	76.4	50.4	87.8	71.1	100									
RB0.05-L	>8	40.2	29.1	17.7	14.6	34.4	100								
RB0.05-L	0.22-8	34.2	14.4	12.9	2.5	38.0	74.3	100							
HP10-L	0.22-8	0.0	0.0	19.9	16.3	14.6	0.0	0.0	100						
HP10-D	0.22-8	0.0	0.0	0.0	0.0	0.0	0.0	0.0	85.8	100					
C-L	0.22-8	16.3	0.0	0.4	0.0	17.0	3.5	19.7	78.2	79.4	100				
C-D	0.22-8	13.5	0.0	8.9	0.0	21.7	3.1	15.7	87.4	81.9	97.1	100			
RB0.2-D	0.22-8	35.6	0.0	16.5	0.0	45.8	17.9	46.0	48.6	43.3	81.5	78.0	100		
RB0.2-L	>8	44.4	29.5	26.4	19.1	39.0	14.6	25.4	39.1	41.6	53.9	55.0	55.9	100	
RB0.2-L	0.22-8	20.3	0.0	7.5	0.0	26.6	12.4	26.9	39.8	42.0	50.6	55.2	58.2	85.0	100

2008

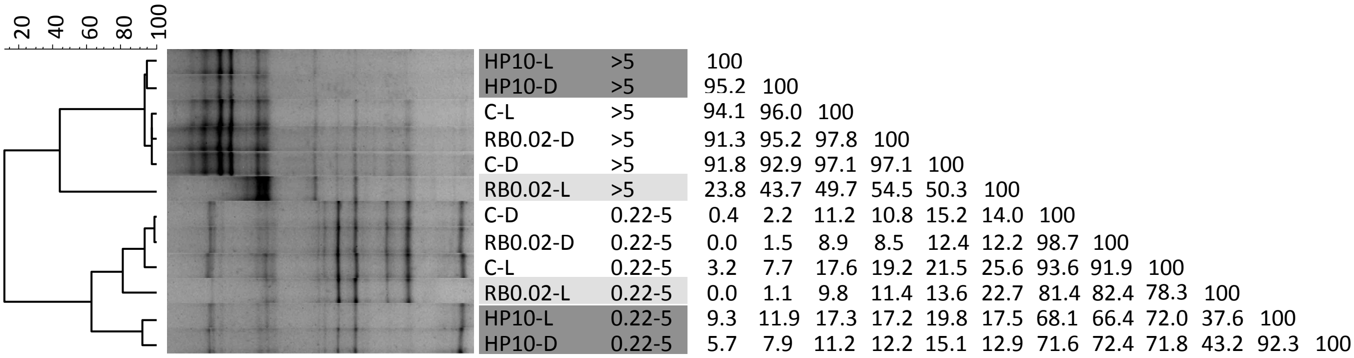

HP10-L	>5	100											
HP10-D	>5	95.2	100										
C-L	>5	94.1	96.0	100									
RB0.02-D	>5	91.3	95.2	97.8	100								
C-D	>5	91.8	92.9	97.1	97.1	100							
RB0.02-L	>5	23.8	43.7	49.7	54.5	50.3	100						
C-D	0.22-5	0.4	2.2	11.2	10.8	15.2	14.0	100					
RB0.02-D	0.22-5	0.0	1.5	8.9	8.5	12.4	12.2	98.7	100				
C-L	0.22-5	3.2	7.7	17.6	19.2	21.5	25.6	93.6	91.9	100			
RB0.02-L	0.22-5	0.0	1.1	9.8	11.4	13.6	22.7	81.4	82.4	78.3	100		
HP10-L	0.22-5	9.3	11.9	17.3	17.2	19.8	17.5	68.1	66.4	72.0	37.6	100	
HP10-D	0.22-5	5.7	7.9	11.2	12.2	15.1	12.9	71.6	72.4	71.8	43.2	92.3	100

2009

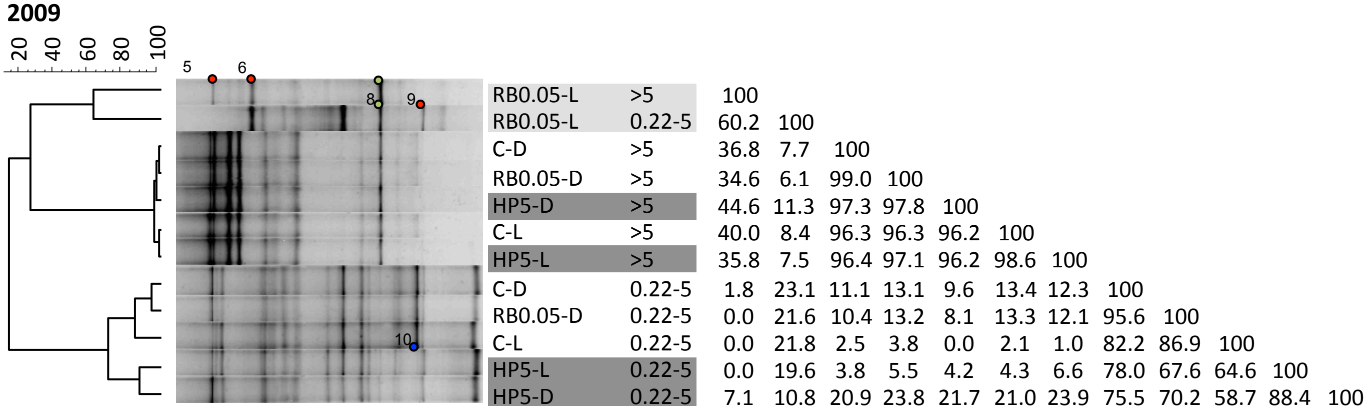

RB0.05-L	>5	100											
RB0.05-L	0.22-5	60.2	100										
C-D	>5	36.8	7.7	100									
RB0.05-D	>5	34.6	6.1	99.0	100								
HP5-D	>5	44.6	11.3	97.3	97.8	100							
C-L	>5	40.0	8.4	96.3	96.3	96.2	100						
HP5-L	>5	35.8	7.5	96.4	97.1	96.2	98.6	100					
C-D	0.22-5	1.8	23.1	11.1	13.1	9.6	13.4	12.3	100				
RB0.05-D	0.22-5	0.0	21.6	10.4	13.2	8.1	13.3	12.1	95.6	100			
C-L	0.22-5	0.0	21.8	2.5	3.8	0.0	2.1	1.0	82.2	86.9	100		
HP5-L	0.22-5	0.0	19.6	3.8	5.5	4.2	4.3	6.6	78.0	67.6	64.6	100	
HP5-D	0.22-5	7.1	10.8	20.9	23.8	21.7	21.0	23.9	75.5	70.2	58.7	88.4	100

Figure S4

Distance matrices of Pearson correlation based UPGMA cluster analysis performed for *Bacteria* RT-PCR DGGE patterns of *in situ* experiments 2006, 2008, and 2009. Abbreviations are given in Figure S2. White numbers on DGGE bands correspond to DGGE band sequences determined in respective PCR-DGGE pattern of the same experiment published previously (Glaeser *et al.*, 2010). Colours indicate the phylogenetic affiliation: *Actinobacteria* (purple), *Gammaproteobacteria* (brown), *Alphaproteobacteria* (red), and *Betaproteobacteria* (blue).
