# Supplementary material for: Contrasting Effects of Singlet Oxygen and Hydrogen Peroxide on Bacterial Community Composition in a Humic Lake
Source: PLoS One. 2014 Mar 25;9(3):e92518. doi: 10.1371/journal.pone.0092518 (PMC3965437; doi:10.1371/journal.pone.0092518)
Supplement: Figure S5 — Robustness of changes in BCC observed by RT-PCR-DGGE analysis shown by repeats of the 2006 (A), 2008 (B) and 2009 (C) in situ experiments. (PDF) [file pone.0092518.s005.pdf]

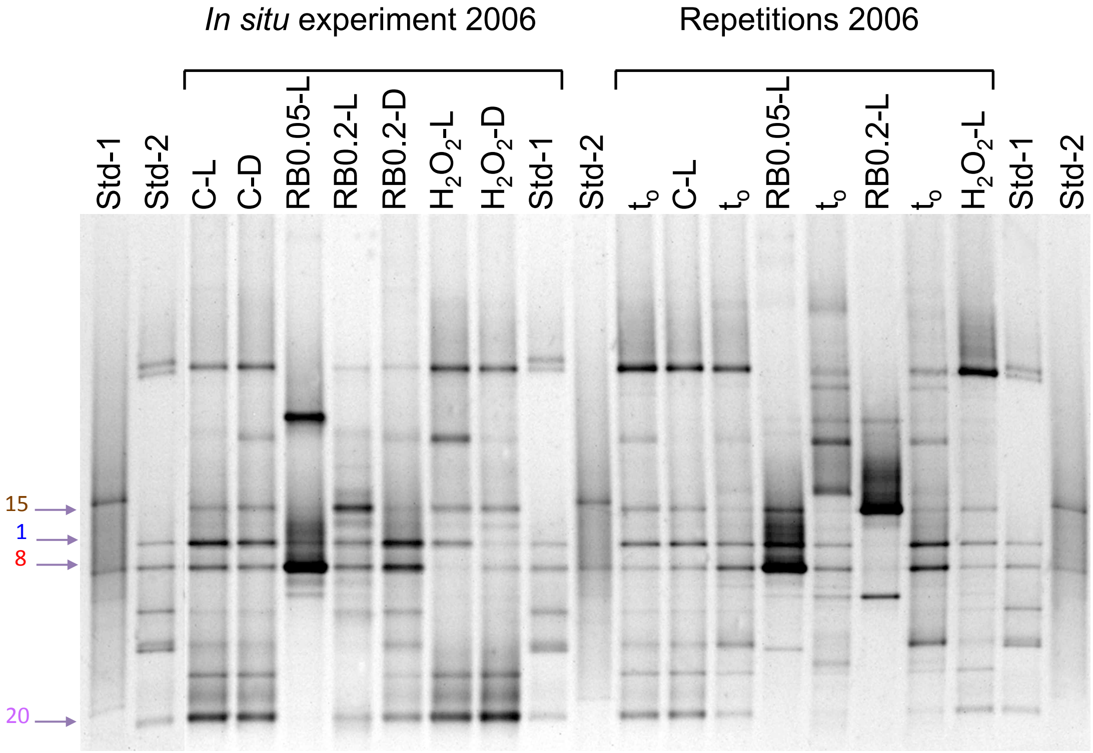

Figure S5

Robustness of changes in BCC observed by RT-PCR-DGGE analysis. *In situ* experiments of 2006, 2008 and 2009 were repeated within a few days before or after the experiments described in the main text. Std-1, Std-2: Mixtures of amplified 16S rRNA gene fragments used as Standards. Abbreviations used in the legends are given in Fig. S2. Std-1, Std-2: Standards.

A. Repeats for the 2006 *in situ* experiment from 12th July 2006 were performed on 14th July (C-L), 15th July (RB0.2-L), 18th July (RB0.05-L) and 20th July (HP-L). In each experiment we compared to a control sample obtained at the start of the experiment (t_0). All days after the 12th of July were sunny without clouds and rain and therefore the weather conditions were highly comparable. To show that the changes in the DGGE pattern were in principle similar to the experiment performed on 12th July the *Bacteria*-RT-PCR DGGE analysis of the free-living bacteria fractions were compared. Colored numbers with arrows represent the phylotypes associated with *P. necessarius* (OTU-1), *N. acidiphilum* (OTU-8), a *Methylococcaceae* species (OTU-15) and an acI-B *Actinobacteria* species (OTU-20). Overall in the repeats similar changes occurred with respect to a BCC that slightly changed over time.

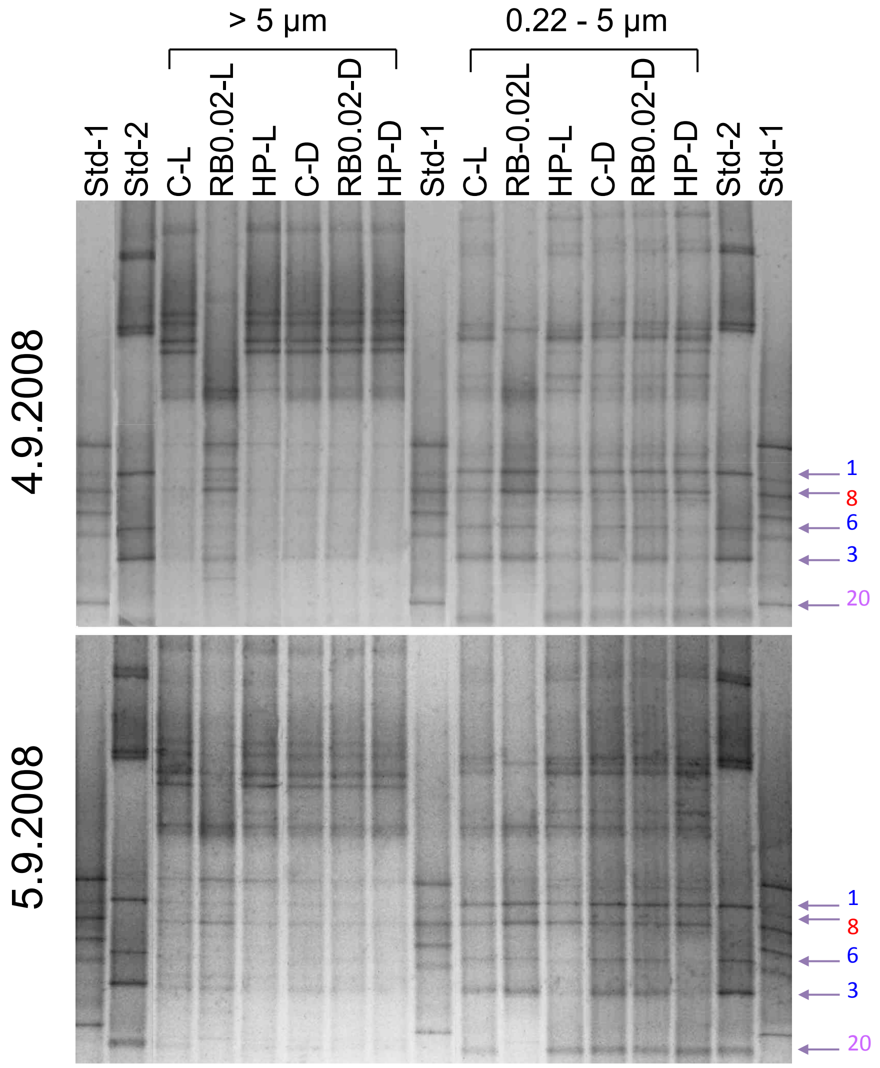

B. In 2008 the whole experiment was performed at two consecutive days (4th and 5th September). Both bacteria fractions were investigated for both days by *Bacteria*-specific RT-PCR DGGE. The patterns and BCC changes were identical for both experiments. Colored numbers with arrows represent the phylotypes associated with *P. necessarius* (OTU-1), *N. acidiphilum* (OTU-8), betI Betaproteobacteria species (OUT-3 and 6), *Methylococcaceae* species (OTU-15) and an acI-B *Actinobacteria* species (OTU-20).

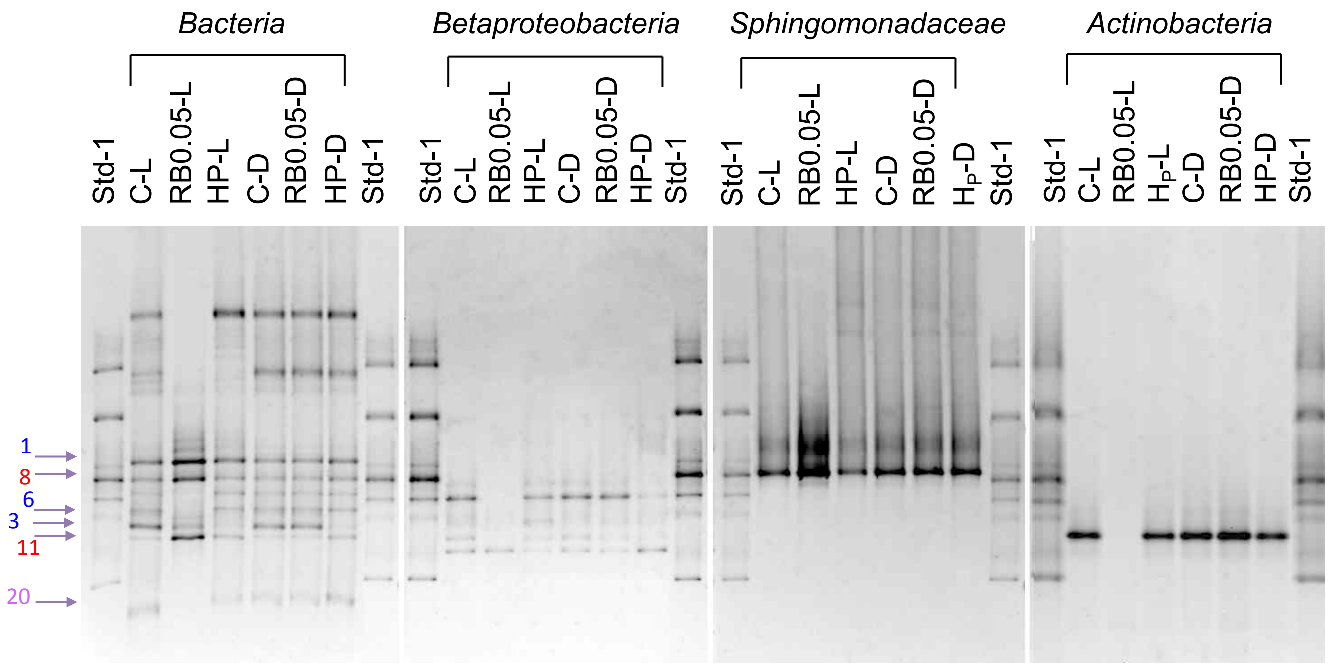

C. The 2009 *in situ* experiment from 14th August was repeated on 17th August. Experimental triplicates were pooled during filtration of the samples and free-living bacteria were investigated with *Bacteria*- and the three group specific primer systems by RT-PCR DGGE analysis. In this figure, only the DGGE patterns for samples obtained in the repetition of the experiment are shown. DGGE pattern for the samples of the experiment form 14th August are depicted in Fig. 10 and 11. DGGE pattern were highly similar for experimental repeats performed within the same year. Changes in the DNA bands representing dominating bacterial phylotypes of the SW basin were highly similar between both experiments, although slight variations were observed, e.g. for RB0.05-L sample analysed with *Betproteobacteria*-specific primers. OTUs represented by predominant DGGE DNA bands that are representative are shown in. Colored numbers with arrows represent the phylotypes associated with *P. necessarius* (OTU-1), betI Betaproteobacteria species (OUT-3 and 6), *N. acidiphilum* (OTU-8), a *Caulobacteraceae* species (OTU-11), and an acI-B *Actinobacteria* species (OTU-20).
