# Supplementary material for: Contrasting Effects of Singlet Oxygen and Hydrogen Peroxide on Bacterial Community Composition in a Humic Lake
Source: PLoS One. 2014 Mar 25;9(3):e92518. doi: 10.1371/journal.pone.0092518 (PMC3965437; doi:10.1371/journal.pone.0092518)
Supplement: Figure S6 — Cluster analysis of Betaproteobacteria, Actinobacteria, and Sphingomonadaceae- specific 16S rRNA gene based RT-PCR DGGE analysis. (PDF) [file pone.0092518.s006.pdf]

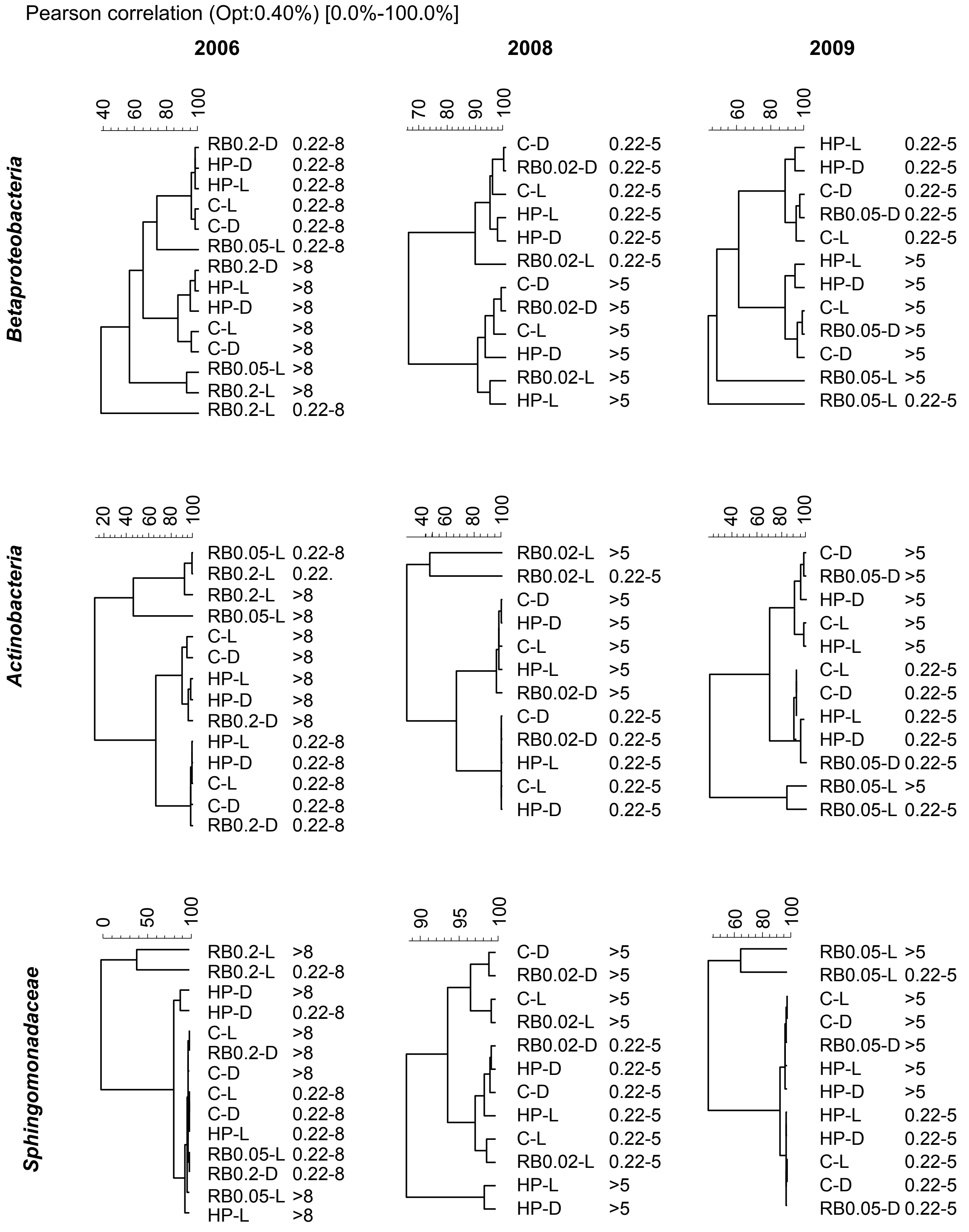

Figure S6

Cluster analysis of *Betaproteobacteria, Actinobacteria,* and *Sphingomonadaceae*-specific 16S rRNA gene based RT-PCR DGGE analysis. Free-living (0.22-8 µm in 2006 and 0.22-5 µm in 2008 and 2009) and particle-attached (>8 or >5 µm, respectively) bacteria of all treatments of the *in situ* experiments 2006, 2008, and 2009 were investigated. The UPGMA cluster analyses based on the Pearson correlation that considers the DNA band intensities. Abbreviations are given in Figure S2.
