# Supplementary material for: Contrasting Effects of Singlet Oxygen and Hydrogen Peroxide on Bacterial Community Composition in a Humic Lake
Source: PLoS One. 2014 Mar 25;9(3):e92518. doi: 10.1371/journal.pone.0092518 (PMC3965437; doi:10.1371/journal.pone.0092518)
Supplement: Figure S7 — Delayed formation of hydrogen peroxide (H2O2) in 0.22 μm filtered water samples exposed to natural sunlight. (PDF) [file pone.0092518.s007.pdf]

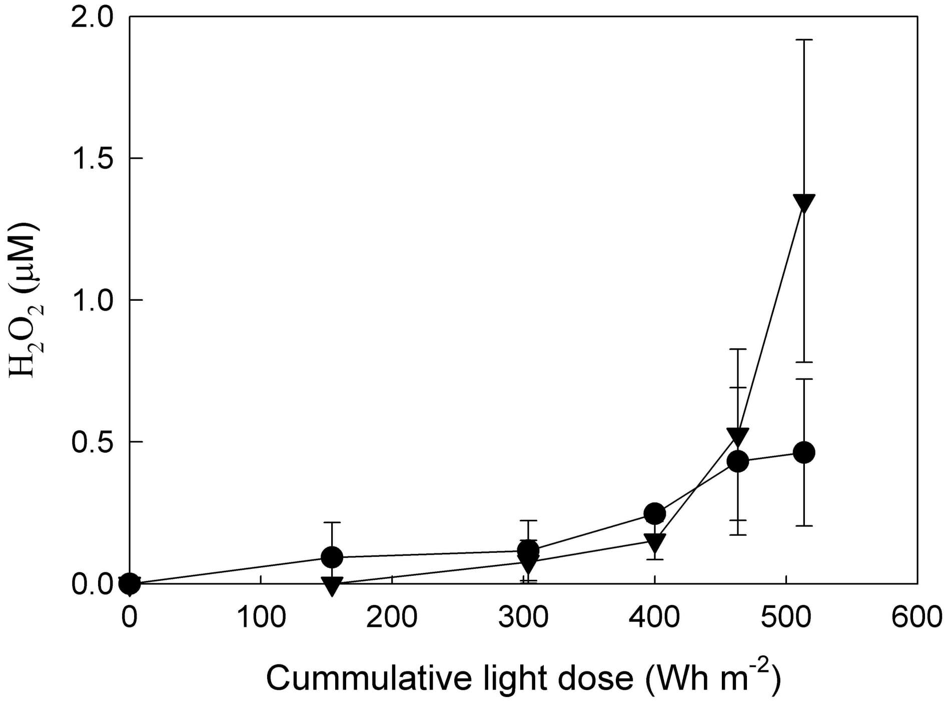

Figure S7
Delayed formation of hydrogen peroxide (H_2O_2) in 0.22 µm filtered water samples exposed to natural sunlight. Samples were used for exposure experiments directly after sampling. Filled circles represent H_2O_2 concentrations filled triangles for samples obtained from wells inside the acidic fen. Water samples were obtained on 2nd September 2008 from the SW basin. Error bars indicate the standard deviation of three analysed samples.
