# Supplementary material for: Contrasting Effects of Singlet Oxygen and Hydrogen Peroxide on Bacterial Community Composition in a Humic Lake
Source: PLoS One. 2014 Mar 25;9(3):e92518. doi: 10.1371/journal.pone.0092518 (PMC3965437; doi:10.1371/journal.pone.0092518)
Supplement: Figure S8 — Transmission scans of poly-propylene (PP) bags, poly-ethylene (PE) Whirl-Pak bags, and the UVA/B block sheet. (PDF) [file pone.0092518.s008.pdf]

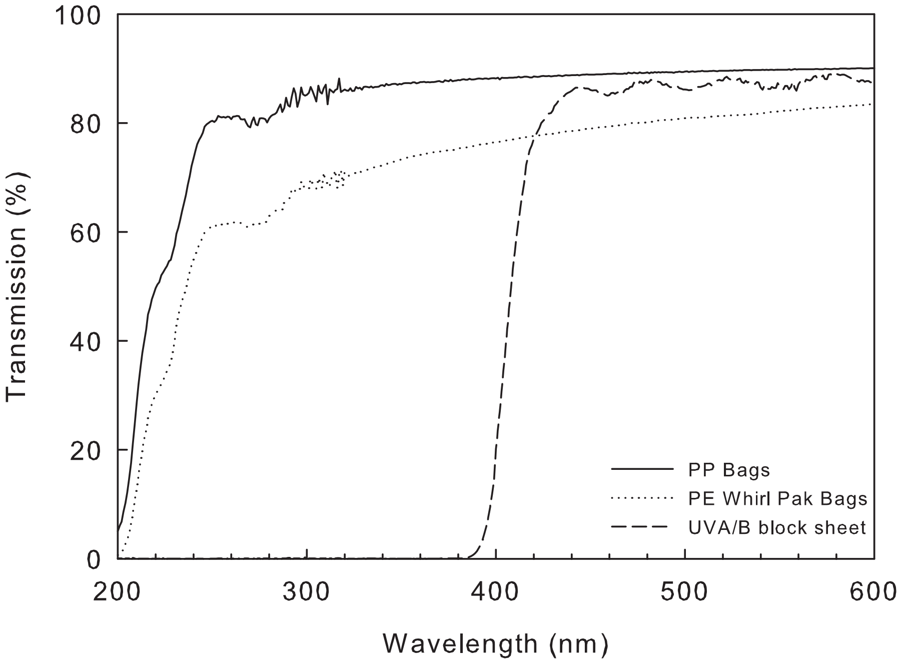

Figure S8

Transmission scans of poly-propylene (PP) bags, poly-ethylene (PE) Whirl-Pak bags, and the UVA/B block sheet. PP and Whirl-Pak bags were purchased from Carl Roth (Karlsruhe, Germany) and the polyester UVA/B block sheet 90 NR from Modulor (Berlin, Germany).
