# Supplementary material for: Contrasting Effects of Singlet Oxygen and Hydrogen Peroxide on Bacterial Community Composition in a Humic Lake
Source: PLoS One. 2014 Mar 25;9(3):e92518. doi: 10.1371/journal.pone.0092518 (PMC3965437; doi:10.1371/journal.pone.0092518)
Supplement: Figure S9 — Weather data for 30 day prior to the experiments carried out in 2006, 2008, and 2009. (PDF) [file pone.0092518.s009.pdf]

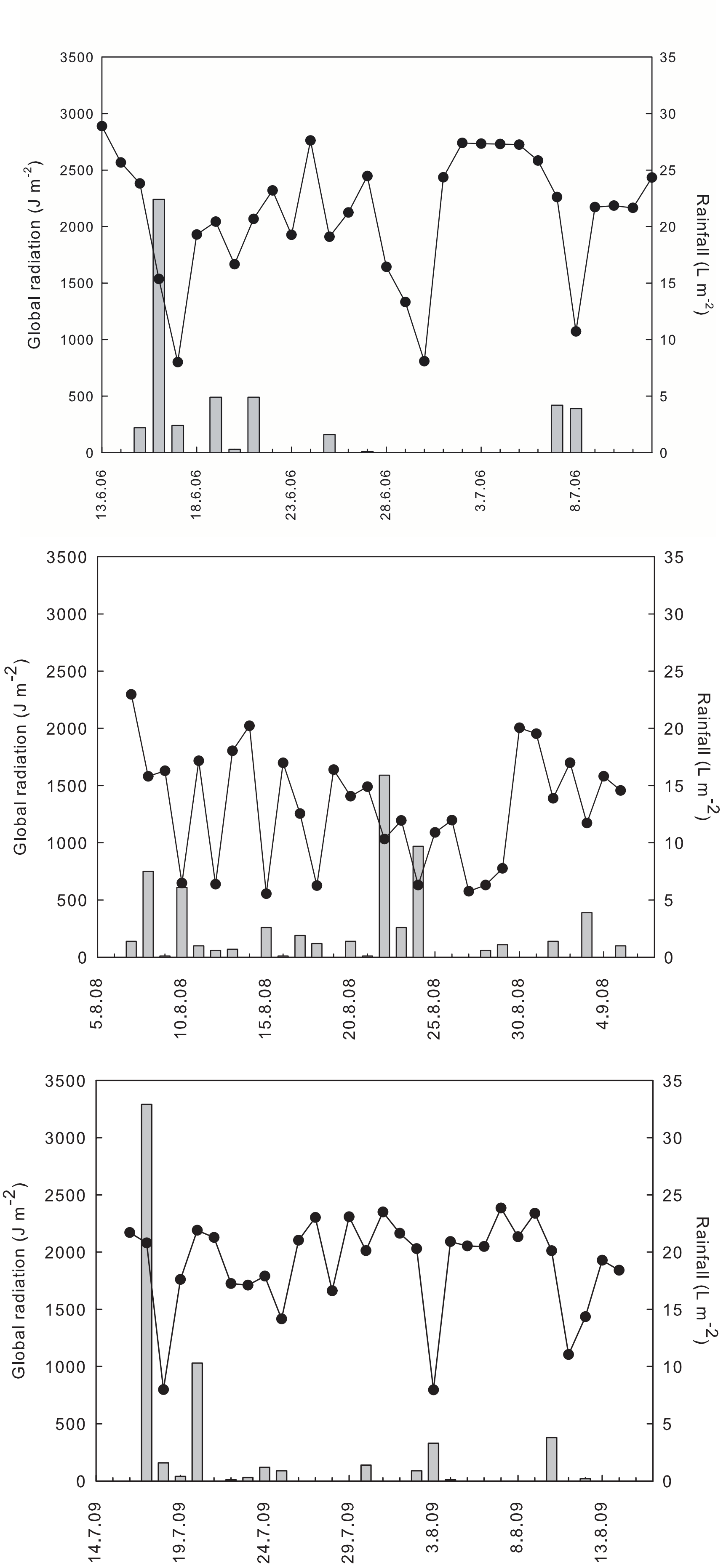

2006
Rain: 46.9 L m^{-2}
Light: 63379 J m^{-2}

2008
Rain: 60.9 L m^{-2}
Light: 39380 J m^{-2}

2009
Rain: 57.4 L m^{-2}
Light: 56870 J m^{-2}:

Figure S9
Weather data for 30 day prior to the experiments carried out in 2006, 2008, and 2009, were obtained at the weather station in Menz (53°10′ N, 13°05′ E), which is closely located to Lake Grosse Fuchskuhle and purchased from the Deutscher Wetterdienst (www.dwd.de). Filled circles: global radiation. Bars: rainfall.
