# Supplementary material for: Contrasting Effects of Singlet Oxygen and Hydrogen Peroxide on Bacterial Community Composition in a Humic Lake
Source: PLoS One. 2014 Mar 25;9(3):e92518. doi: 10.1371/journal.pone.0092518 (PMC3965437; doi:10.1371/journal.pone.0092518)
Supplement: Table S1 — NOM concentrations and reactivity of surface water samples from the SW basin of Lake Grosse Fuchskuhle. (PDF) [file pone.0092518.s010.pdf]

Table S1

NOM concentrations and reactivity of surface water samples from the SW basin of Lake Grosse Fuchskuhle. Characteristics of 0.22 µm filtered water samples are compiled with respect to their reactivity and capacity to form singlet oxygen ($^1O_2$) and hydrogen peroxide (H_2O_2). UV-A/B: irradiation with 50 W m^{-2} of UV-A and 1 W m^{-2} UV-B (Helarium B1-12-40W, Wolff System). SW: South-West basin of Lake Grosse Fuchskuhle, AFW: Acidic Fen Water.

**Parameter**	**Samples**			
	**2006 SW**	**2008 SW**	**2009 SW**	**2008 AFW**
**DOC (mg C L^{-1})**	23.3 ± 1.8	34.0 ± 0.1	28.4 ± 1.1	19.3 ± 0.3
**A_{254}**	0.78	1.27	1.21	0.54
**$SUVA_{254}$ (L mg^{-1} m^{-1})$^+$**	3.3	3.7	4.3	2.8
**$[^1O_2]_{SS}$ UV-A/B (10^{-14} M)**	16.7 ± 1.5	18.8 ± 0.3	20.8 ± 1.5	12.9 ± 0.1
**$[^1O_2]_{SS}$ UV-A/B (10^{-14} M mg^{-1} DOC^{-1} L^{-1})**	0.71	0.55	0,73	0,67
**H_2O_2 UV-A/B (µM h^{-1})**	0.23 ± 0.04	0.35 ± 0.07	1.04 ± 0.08	1.43 ± 0.35
**H_2O_2 UV-A/B (µM h^{-1} mg^{-1} DOC^{-1} L^{-1})**	9.3 x 10^{-3}	10.3 x 10^{-3}	36.6 x 10^{-3}	74 x 10^{-3}
**Ratio: H_2O_2 (µM h^{-1}) / $[^1O_2]_{SS}$ (10^{-14} M) × 1000**	13.8	18.6	50	110

$^+$: $SUVA_{254}$: specific UVA absorbance at 254 nm; Determined after [1]

n.d.: not determined

Reference:

1. Weishaar JL, Aiken GR, Bergamaschi BA, Fram MS, Fujii R, et al. (2003) Evaluation of specific ultraviolet absorbance as an indicator of the chemical composition and reactivity of dissolved organic carbon. Environ Sci Technol 37: 4702-4708.
