# Supplementary material for: Contrasting Effects of Singlet Oxygen and Hydrogen Peroxide on Bacterial Community Composition in a Humic Lake
Source: PLoS One. 2014 Mar 25;9(3):e92518. doi: 10.1371/journal.pone.0092518 (PMC3965437; doi:10.1371/journal.pone.0092518)
Supplement: Table S2 — Cell numbers of in situ incubation experiments in 2006 and 2008. (PDF) [file pone.0092518.s011.pdf]

Table S2

Cell numbers of *in situ* incubation experiments in 2006 and 2008. Cell counts of Sybr Green I stained cells collected on 0.22 µm membrane filters. Standard deviations of ten counts from each experiment are given in brackets. Cell counts were divided into cells with length of >0.35 µm and <0.35 µm (representing micrococcoid cells). C-L/D: Control of light and dark incubations, RB-L: treatments with increased $[^1O_2]_{SS}$; numbers indicate µM concentrations of RB added, RB-D: Dark control of RB treatments, HP10-L/D: Light and dark incubations with 10 µM H_2O_2.

Experiment	Total [×10^6 cells mL^{-1}]	>0.35 µm [×10^6 cells mL^{-1}]	<0.35 µm Micrococcoids [×10^6 cells mL^{-1}]	Fraction of <0.35 µm cells (%)
***In situ* experiment 2006 (02. July)**				
C-L	3.71 (±0.3)	1.85 (±0.22)	1.86 (±0.19)	50.1 (±2.9)
C-D	4.71 (±1.1)	2.14 (±0.70)	2.57 (±0.84)	54.5 (±12.7)
RB0.05-L	3.72 (±0.58)	1.03 (±0.19)	2.69 (±0.44)	72.4 (±3.2)
RB0.2-L	4.45 (±0.54)	1.76 (±0.24)	2.68 (±0.51)	60.3 (±8.7)
RB0.2-D	4.08 (±0.32)	2.31 (±0.19)	1.77 (±0.26)	43.4 (±5.5)
HP10-L	4.00 (±0.50)	1.71 (±0.25)	2.29 (±0.35)	57.2 (±5.1)
HP10-D	4.95 (±0.45)	2.25 (±0.30)	2.70 (±0.52)	54.6 (±9.3)
***In situ* experiment 2008 (5. September)**				
C-L	2.06 (±0.18)	0.51 (±0.10)	1.55 (±0.17)	75.1 (±5.2)
C-D	1.30 (±0.08)	0.45 (±0.04)	0.85 (±0.06)	65.2 (±2.4)
RB0.02-L	3.37 (±0.53)	0.47 (±0.06)	2.90 (±0.52)	86.1 (±7.0)
RB0.02-D	2.00 (±0.10)	0.49 (±0.07)	1.51 (±0.10)	75.4 (±3.4)
HP10-L	1.36 (±0.24)	0.41 (±0.06)	0.94 (±0.21)	69.5 (±9.4)
HP10-D	1.36 (±0.16)	0.39 (±0.05)	0.97 (±0.15)	71.2 (±7.5)
