# Supplementary material for: Contrasting Effects of Singlet Oxygen and Hydrogen Peroxide on Bacterial Community Composition in a Humic Lake
Source: PLoS One. 2014 Mar 25;9(3):e92518. doi: 10.1371/journal.pone.0092518 (PMC3965437; doi:10.1371/journal.pone.0092518)
Supplement: Table S3 — Physico-chemical parameters of Lake Grosse Fuchskuhle SW compartment. (PDF) [file pone.0092518.s012.pdf]

Table S3
Physico-chemical parameters measured in surface waters of Lake Grosse Fuchskuhle SW basin during the experimental periods in 2006, 2008, and 2009. Data for pH, temperature and oxygen were monitored according to [1].

**Parameter**	**Lake Grosse Fuchskuhle**
Geographical position	53°10´N, 13°02´E[1]
Maximum depth (m)	5.6[1]
Surface area (km^2)	0.02[1]
Volume (10^6 m^3)	0.05[1]
Catchment area (km^2)	0.005[1]
Characteristics of catchment area	Mixed forest, fen area[1]
Trophy	Dystrophic[1]

	**SW surface water**		
Year	2006[2]	2008[3]	2009[4]
pH	4.65	4.55	4.5
Temperature (°C)	23.9	17.4	19
Oxygen (mg L^{-1})	4.3	4.8	4.6

[1] Data taken from [2, 3][2] 13th July 2006; [3] 01st September 2008; [4] 19th August 200

References:

1. Glaeser J, Overmann J (2003) Characterization and *in situ* carbon metabolism of phototrophic consortia. Appl Environ Microbiol 69: 3739-3750.
2. Allgaier M, Grossart H-P (2006) Seasonal dynamics and phylogenetic diversity of free-living and particle-associated bacterial communities in four lakes in northeastern Germany. Aquat Microb Ecol 45: 115-128.
3. Allgaier M, Grossart H-P (2006) Diversity and seasonal dynamics of Actinobacteria populations in four lakes in northeastern Germany. Appl Environ Microbiol 72: 3489-3497.
