# Supplementary material for: Contrasting Effects of Singlet Oxygen and Hydrogen Peroxide on Bacterial Community Composition in a Humic Lake
Source: PLoS One. 2014 Mar 25;9(3):e92518. doi: 10.1371/journal.pone.0092518 (PMC3965437; doi:10.1371/journal.pone.0092518)
Supplement: Table S4 — Sequences of 16S rRNA gene targeting oligonucleotide primer used for RT-PCR DGGE analysis. (PDF) [file pone.0092518.s013.pdf]

Table S4
Sequences of 16S rRNA gene targeting oligonucleotide primer used for RT-PCR DGGE analysis. Sequences for the GC clamps are given in the footnotes 2 and 3.

Primer	Target group	Sequence (5´-> 3´)	Reference
GC-339F[2]	*Bacteria*	CTCCTACGGGAGGCAGCAG	[1]
GC-517F[2]	*Bacteria*	GTGCCAGCAGCCGCGG	[2]
907R	*Bacteria*	CCGTCAATTCCTTTGAGTTT	[1]
GC-1055R[2]	*Bacteria*	AGCTGACGACAGCCAT	[3]
GC-Beta359F[2]	*Betaproteobacteria*	GGGGAATTTTGGACAATGGG	[4]
Beta680F	*Betaproteobacteria*	CRCGTGTAGCAGTGA	[5]
Beta680R	*Betaproteobacteria*	TCACTGCTACACGYG	[5]
GC-HGC236F[3]	*Actinobacteria*	GCGGCCTATCAGCTTGTT	[6]
HGC664R	*Actinobacteria*	AGGAATTCCAGTCTCCCC	[6]
ADF681R	*Alphaprotobacteria, some Deltaproteobacteria and Fusobacteria*	AATTTCACCTCTACACT	[5]
ALF968R	*Alphaproteobacteria*	GGTAAGGTTCTGCGCGTT	[7]
Sphingo108F[2]	*Sphingomonadaceae*	GCGTAACGCG TGGGAATCTG	[8]

F: forward primer R: reverse primer
[1] The numbering of the positions is according to the 16S rRNA gene sequences of the *rrnB* operon of *E. coli* (J01695) [9]
[2] GC clamp sequence: CGCCCGCCGCGCCCCGCGCCCGGCCCGCCGCCCCCGCCCC
[3] GC clamp sequence: CGCCCGCCGCGCCCCGCGCCCGTCCCGCCGCCCCCGCCCG

References

1. Muyzer G, de Waal EC, Uitterlinden AG (1993) Profiling of complex microbial populations by denaturing gradient gel electrophoresis analysis of polymerase chain reaction-amplified genes coding for 16S rRNA. Appl Environ Microbiol 59: 695-700.
2. Overmann J, Coolen MJL, Tuschak C (1999) Specific detection of different phylogenetic groups of chemocline bacteria based on PCR and denaturing gradient gel electrophoresis of 16S rRNA gene fragments. Arch Microbiol 172: 83-94.
3. Lane DJ (1991) 16S/23S rRNA sequencing. In: Stackebrandt E, Goodfellow M, editors. Nucleic acid techniques in bacterial systematics. Chichester: John Wiley. pp. 115-174.
4. Mühling M, Woolven-Allen J, Murrell JC, Joint I (2008) Improved group-specific PCR primers for denaturing gradient gel electrophoresis analysis of the genetic diversity of complex microbial communities. ISME J 2: 379-392.
5. Blackwood CB, Oaks A, Buyers JS (2005) Phylum- and class-specific PCR primers for general microbial community analysis. Appl Environ Microbiol 71: 6193-6198.

6. Glöckner FO, Zaichikov E, Belkova N, Denissova L, Pernthaler J, et al. (2000) Comparative 16S rRNA analysis of lake bacterioplankton reveals globally distributed phylogenetic clusters including an abundant group of *Actinobacteria*. Appl Environ Microbiol 66: 5053-5065.
7. Neef A (1997) Anwendung der *in situ*-Einzelzell-identifizierung von Bakterien zur Populationsanalyse in komplexen mikrobiellen Biozönosen. Dissertation, Technical University of Munich.
8. Leys NM, Ryngaert A, Bastiaens L, Top EM, Verstraete W, et al. (2005) Culture independent detection of *Sphingomonas* sp EPA 505 related strains in soils contaminated with polycyclic aromatic hydrocarbons (PAHs). Microb Ecol 49: 443-450.
9. Brosius J, Palmer ML, Kennedy PJ, Noller HF (1978) Complete nucleotide-sequence of a 16S ribosomal-RNA gene from *Escherichia coli*. Proc Nat Acad Sci USA 75: 4801-4805.
