# Supplementary material for: Contrasting Effects of Singlet Oxygen and Hydrogen Peroxide on Bacterial Community Composition in a Humic Lake
Source: PLoS One. 2014 Mar 25;9(3):e92518. doi: 10.1371/journal.pone.0092518 (PMC3965437; doi:10.1371/journal.pone.0092518)
Supplement: Table S5 — PCR and RT-PCR programs for the amplification of 16S rRNA and 16S rRNA gene fragments of Bacteria and bacterial subgroups used for DGGE fingerprint analysis. (PDF) [file pone.0092518.s014.pdf]

Table S5
PCR and RT-PCR programs for the amplification of 16S rRNA and 16S rRNA gene fragments of *Bacteria* and bacterial subgroups used for DGGE fingerprint analysis. All RT-PCR programs started with a reverse transcriptase step of 30 min at 50°C followed by a step for reverse transcriptase inactivation and *Taq*-DNA polymerase activation at 95°C for 15 min. All RT-PCR programs finished with a final elongation step at 72°C. This step was elongated to 30 min to prevent the formation of DNA double bands during DGGE analysis [1].

**Target group**	***Bacteria***	***Betaproteobacteria***	***Actinobacteria***	***Sphingomonadaceae***
Primer-system	GC-339F<br>907R	GC-Beta359F<br>Beta680R	GC-HGC236F<br>HGC664R	Sphingo108F<br>ADF681R<br>Nested-PCR:<br>GC-339F/907R
Fragment length (nt)	588 (+40)	336 (+40)	428 (+40)	871<br>Nested-PCR: 588 (+40)
PCR cycles	94°C 30 sec<br>55°C 30 sec<br>72°C 40 sec<br>28 cycles	94°C 30 sec<br>52°C 30 sec<br>72°C 35 sec<br>28-30 cycles	94°C 30 sec<br>52°C 30 sec<br>72°C 35 sec<br>28 cycles	94°C 30 sec<br>55°C 40 sec<br>72°C 54 sec<br>28 cycles

Reference

1. Janse I, Bok J, Zwart G (2004) A simple remedy against artifactual double bands in denaturing gradient gel electrophoresis. J Microbiol Met 57: 279-281.
