# Supplementary material for: Contrasting Effects of Singlet Oxygen and Hydrogen Peroxide on Bacterial Community Composition in a Humic Lake
Source: PLoS One. 2014 Mar 25;9(3):e92518. doi: 10.1371/journal.pone.0092518 (PMC3965437; doi:10.1371/journal.pone.0092518)
Supplement: Table S6 — Phylogenetic affiliation of 16S rRNA gene sequences representing DGGE bands. (PDF) [file pone.0092518.s015.pdf]

Table S6

Phylogenetic affiliation of 16S rRNA gene sequences representing DGGE bands. Phylogenetic assignments of 16S rRNA gene sequences were performed by using the RDP II Classifier [1] and BLAST [2]. DGGE bands were derived from DGGE patterns generated with *Bacteria* (**EUB**), *Betaproteobacteria* (**Beta**), *Actinobacteria* (**Actino**), or *Sphingomonadaceae* (**SPH**)-specific 16S rRNA gene targeting primer-systems. SW: **S**outh-**W**est basin of Lake Grosse Fuchskuhle; **06**, **08**, or **09**: experiments in 2006, 2008, and 2009. Sequence names correspond to those shown in the phylogenetic trees of Fig. 5-9 and the numbers within the sequence names correspond to numbers next to DNA bands in the respective DGGE gels.

Sequence Name	Acc. No.	nt	RDP classifier	Closest related BLAST hit		
				**Similarity (%)**	**Acc.**	**Sequence name**
EUB-SW06-1	JF917134	365	*"Chlorobi"[100%] "Chlorobia"[100%] Chlorobiales[100%] Chlorobiaceae[100%] Chlorobium[100%]*	100%<br>99%	GU734037<br>CP001110	Lake Grosse Fuchskhuhle DGGE band FSW-St05-2<br>*Pelodictyon phaeoclathratiforme* BU-1
EUB-SW06-2	JF917135	374	*Alphaproteobacteria[95%] Rhizobiales[46%]*	100%<br>95%	HQ420131<br>EF667926	Acidic cole mine drainage clone LRE22B42<br>Uncultured alpha proteobacterium clone Hv(lakePohlsee)_38
EUB-SW06-3	JF917136	388	*Gammaproteobacteria[100%] Oceanospirillales[47%] Oleiphilaceae[42%] Oleiphilus[42%]*	99%<br>93%	GU734062<br>AE017282	Lake Grosse Fuchskuhle DGGE band FSW-St06-11<br>*Methylococcus capsulatus* Bath
EUB-SW06-4	JF917137	452	*Alphaproteobacteria[100%] Sphingomonadales[100%] Sphingomonadaceae[100%] Novosphingobium[100%]*	99%	EU336977	*N. acidiphilum* FSW06-204d^T
EUB-SW09-5	JF917138	408	*Cyanobacteria[100%] Cyanobacteria[100%] Chloroplast[100%] Chlorophyta[100%]*	100%	EU442895	Nam Co Lake clone N06Jun-31
EUB-SW09-6	JF917139	505	*Alphaproteobacteria[99%] Rhizobiales[61%] Bradyrhizobiaceae[20%] Balneimonas[19%]*	95%	EF667926<br>HM68944	Lake Pohlsee clone Hv(lakePohlsee)_38<br>Acidic mining lake DGGE band B3
EUB-SW09-7	JF917140	367	*Firmicutes"[100%] "Bacilli"[98%] Bacillales[98%] "Paenibacillaceae"[87%] Paenibacillus[87%]*	100%	GU734065	Lake Grosse Fuchskuhle DGGE band FSW-St06-14
EUB-SW09-8	JF917141	532	*Firmicutes"[100%] "Bacilli"[100%] Bacillales[100%] "Paenibacillaceae"[89%] Paenibacillus[88]*	99%	GU734065	Lake Grosse Fuchskuhle DGGE band FSW-St06-14
EUB-SW09-9	JF917142	505	*Alphaproteobacteria[100%]*	98%	EF520395	Adirondack lake clone ADK-BTe02-51

			Caulobacterales[100%] Caulobacteraceae[100%]<br>*Phenylobacterium[86%]*			
EUB-SW09-10	JF917143	530	*Betaproteobacteria[100%]*<br>*Burkholderiales[100%] Comamonadaceae[98%]*<br>*Acidovorax[34%]*	99%	GU734044<br>DQ501302	Lake Grosse Fuchskuhle DGGE band FSW-St05-9<br>Lake Grosse Fuchskuhle clone FNE11-10
Beta-SW06-1	JF917144	285	*Betaproteobacteria[100%]*<br>*Burkholderiales[100%] Comamonadaceae[98%]*<br>*Simplicispira[58%]*	100%<br>99%<br>95%	GU734058<br>EF520440<br>AF435948	Lake Grosse Fuchskuhle DGGE band FSW-St06-7<br>Adriondack Lake clone ADK-BMe02-21<br>*Rhodoferax ferrireducens* T118^T
Beta-SW06-2	JF917145	287	*Betaproteobacteria[100%]*<br>*Burkholderiales[100%] Burkholderiaceae[100%]*<br>*Polynucleobacter[100%]*	100%	AJ879783	*P. necessarius* ssp. *asymbioticus* QLW-P1DMWA-1^T
Beta-SW06-3	JF917146	279	*Betaproteobacteria[100%]*<br>*Burkholderiales[100%]*<br>*Comamonadaceae[100%] Simplicispira[14%]*	100%<br>99%<br>96%	GU734044<br>DQ501302<br>AF285414	Lake Grosse Fuchskuhle DGGE band FSW-St05-9<br>Lake Grosse Fuchskuhle clone FNE11-10<br>Xenophilus azovorans KF46F^T
Beta-SW06-4	JF917147	276	*Betaproteobacteria[100%]*<br>*Methylophilales[100%] Methylophilaceae[100%]*<br>*Methylophilus[99%]*	99%<br>96%	GU734054<br>GQ175365	Lake Grosse Fuchskuhle DGGE band FSW06-St06-3<br>*Methylophilus* sp. Tobacco-planted soil isolate CS3
Beta-SW06-5	JF917148	281	*Betaproteobacteria[100%]*<br>*Burkholderiales[100%]*<br>*Comamonadaceae[100%] Curvibacter[56%]*	99%<br>96%	GU734088<br>AB109889	Lake Grosse Fuchskuhle DGGE band FSW06-Lt06-1<br>*Curvibacter gracilis* 7-1^T
Beta-SW06-6	JF925282	173	*Betaproteobacteria[100%]*<br>*Burkholderiales[100%] Burkholderiaceae[100%]*<br>*Polynucleobacter[100%]*	100%	AJ879783<br>GU734064	*P. necessarius* ssp. *asymbioticus* QLW-P1DMWA-1^T<br>Lake Grosse Fuchskuhle DGGE band FSW-St06-13
Beta-SW08-7	JF917149	277	*Betaproteobacteria[100%]*<br>*Burkholderiales[100%] Burkholderiaceae[54%]*<br>*Polynucleobacter[54%]*	99%<br>98%	HM128455<br>FM208182	Tanggulha Lake clone SINN1071<br>*Polynucleobacter rarus* MT-CBb6A5^T
Beta-SW08-8	JF917150	285	*Betaproteobacteria[100%]*<br>*Burkholderiales[99%] Comamonadaceae[99%]*<br>*Simplicispira[45%]*	100%<br>99%<br>95%	GU734058<br>EF520440<br>AF435948	Lake Grosse Fuchskuhle DGGE band FSW-St06-7<br>Adriondack Lake clone ADK-BMe02-21<br>*Rhodoferax ferrireducens* T118^T
Beta-SW08-9	JF917151	279	*Betaproteobacteria[100%]*<br>*Burkholderiales[100%] Comamonadaceae[99%]*<br>*Simplicispira[24%]*	100%<br>99%<br>96%	GU734044<br>DQ501302<br>AF285414	Lake Grosse Fuchskuhle DGGE band FSW-St05-9<br>Lake Grosse Fuchskuhle clone FNE11-10<br>Xenophilus azovorans KF46F^T
Beta-SW09-10	JF917152	292	*Betaproteobacteria[100%]*<br>*Burkholderiales[100%]*<br>*Comamonadaceae[100%] Simplicispira[61%]*	100%<br>99%<br>95%	GU734058<br>EF520440<br>AF435948	Lake Grosse Fuchskuhle DGGE band FSW-St06-7<br>Adriondack Lake clone ADK-BMe02-21<br>*Rhodoferax ferrireducens* T118^T
Beta-SW09-11	JF917153	271	*Betaproteobacteria[100%]*<br>*Burkholderiales[100%] Burkholderiaceae[100%]*<br>*Polynucleobacter[100%]*	100%	AJ879783	*P. necessarius* ssp. *asymbioticus* QLW-P1DMWA-1^T

Actino-SW06-1	JF917154	421	*Actinobacteria[92%] Actinobacteridae[89%] Actinomycetales[85%] Micrococcineae[47%] Intrasporangiaceae[26%] Intrasporangium[6%]*	99%	DQ316352	Lake Grosse Fuchskuhle clone FSW11-20
Actino-SW06-2	JF917155	421	*Actinobacteria[99%] Actinobacteridae[97%] Actinomycetales[90%] Micrococcineae[52%] Intrasporangiaceae[32%] Terracoccus[5%]*	100%<br>99%	EU117631<br>DQ316352	Crystal Bog Lake clone CB7B1<br>Lake Grosse Fuchskuhle clone FSW11-20
Actino-SW06-3	JF917156	402	*Verrucomicrobia"[100%] Subdivision3[100%] Subdivision3_genera_incertae_sedis[100%]*	98%	AY792311	Crystal Bog Lake clone CystalBoq022E6
Actino-SW06-4	JF917157	388	*Actinobacteria"[94%] Actinobacteria[94%] Actinobacteridae[88%] Actinomycetales[86%] Micrococcineae[49%] Intrasporangiaceae[30%] Terracoccus[6%]*	100%<br>99%	HQ532266<br>DQ316348	Crystal Bog Lake clone CB3D11<br>Lake Grosse Fuchskuhle clone FSW11-16
Actino-SW06-5	JF917158	402	*"Verrucomicrobia"[100%] Subdivision3[100%] Subdivision3_genera_incertae_sedis[100%]*	98%	FJ624906	Boreal pine forest clone HF_C_33
Actino-SW06-6	JF917159	400	*Verrucomicrobia"[100%] Subdivision3[100%] Subdivision3_genera_incertae_sedis[100%]*	99%	AY792311	Crystal Bog Lake clone CystalBoq022E6
Actino-SW06-7	JF917160	419	*"Verrucomicrobia"[100%] Spartobacteria[98%] Spartobacteria_genera_incertae_sedis[96%]*	97%	FJ916701	Twin Valley Lake clone TW1A5
Actino-SW08-8	JF917161	380	*Actinobacteria[100%] Actinobacteridae[100%] Actinomycetales[100%] Corynebacterineae[100%] Mycobacteriaceae[100%] Mycobacterium[100%]*	100%<br>98%	FJ544445<br>AY208856	*Mycobacterium* sp. S061<br>*Mycobacterium saskatchewanense* NRCM 00-250^T
Actino-SW08-9	JF917162	414	*"Verrucomicrobia"[100%] Subdivision3[100%] Subdivision3_genera_incertae_sedis[100%]*	98%	AY792311	Crystal Bog Lake clone CystalBoq022E6
Actino-SW08-10	JF917163	414	*"Verrucomicrobia"[100%] Subdivision3[100%] Subdivision3_genera_incertae_sedis[100%]*	98%<br>97%	HQ162248<br>AY792311	Arctic peat soil clone Jun20<br>Crystal Bog Lake clone CystalBoq022E6
Actino-SW08-11	JF917164	402	*"Verrucomicrobia"[100%] Subdivision3[100%] Subdivision3_genera_incertae_sedis[100%]*	98%	AY792311	Crystal Bog Lake clone CystalBoq022E6
SPH-SW06-1	JF917165	508	*Alphaproteobacteria[100%] Rhodospirillales[100%] Acetobacteraceae[100%] Kozakia[24%]*	99%	GU734042	Lake Grosse Fuchskuhle DGGE bands FSW-St05-7
SPH-SW08-2	JF917166	505	*Alphaproteobacteria[100%] Rhodospirillales[100%] Rhodospirillaceae[100%] Telmatospirillum[100%]*	99%<br>97%	GQ402757<br>AF524863	Peat swamp forest soil clone clone PW351<br>*Telmatosphirillum siberiense* 26-4b1
SPH-SW08-3	JF917167	505	*Alphaproteobacteria[100%] Rhodospirillales[100%] Rhodospirillaceae[98%] Skermanella[81%]*	94%	GU256437	Azospirillum amazonense DSM 2787^T
SPH-SW08-4	JF917168	505	*Alphaproteobacteria[100%] Sphingomonadales[100%]*	99%	EU336977	*N. acidiphilum* FSW06-204d^T

			Sphingomonadaceae[100%]			
			Novosphingobium[100%]			
SPH-SW08-5	JF917169	505	*Alphaproteobacteria[100%]*	96%	AF524863	*Telmatosphirillum siberiense* 26-4b1
			Rhodospirillales[100%]			
			Rhodospirillaceae[100%]			
			Telmatospirillum[99%]			
SPH-SW08-6	JF917170	503	*Alphaproteobacteria[100%]*	99%	EU409478	Lake Grosse Fuchskuhle isolate Fuku2-ISO-153
			Rhodospirillales[98%] Rhodospirillaceae[78%]	94%	GU256437	*Azospirillum amazonense* DSM 2787^T
			Rhodocista[33%]			
SPH-SW08-7	JF917171	506	*Alphaproteobacteria[100%]*	94%	GU256437	Azospirillum amazonense DSM 2787T
			Rhodospirillales[100%] Rhodospirillaceae[99%]			
			Skermanella[77%]			
SPH-SW09-8	JF917172	505	*Alphaproteobacteria[100%]*	99%	GQ402757	Peat swamp forest soil clone clone PW351
			Rhodospirillales[100%]	97%	AF524863	*Telmatosphirillum siberiense* 26-4b1
			Rhodospirillaceae[100%]			
			Telmatospirillum[100%]			
SPH-SW09-9	JF917173	505	*Alphaproteobacteria[100%]*	95%	GU256437	*Azospirillum amazonense* DSM 2787^T
			Rhodospirillales[100%] Rhodospirillaceae[99%]			
			Skermanella[79%]			
SPH-SW08-10	JF917174	503	*Alphaproteobacteria[100%]*	99%	GQ402757	Peat swamp forest soil clone PW351
			Rhodospirillales[100%]	97%	AF524863	*Telmatosphirillum siberiense* 26-4b1
			Rhodospirillaceae[100%]			
			Telmatospirillum[98%]			
SPH-SW09-11	JF917175	495	*Gammaproteobacteria[99%]*	94%		*Methylobacter*
			Methylococcales[96%] Methylococcaceae[96%]	93%	AJ290055	Lake Grosse Fuchskuhle clone Fuku N33
			Methylobacter[87%]			
SPH-SW09-12	JF917176	506	*Alphaproteobacteria[100%]*	99%	EU336977	*N. acidiphilum* FSW06-204d^T
			Sphingomonadales[100%]			
			Sphingomonadaceae[100%]			
			Novosphingobium[100%]			
SPH-SW09-13	JF917177	498	*Alphaproteobacteria[100%]*	99%	EU336977	*N. acidiphilum* FSW06-204d^T
			Sphingomonadales[100%]			
			Sphingomonadaceae[99%]			
			Novosphingobium[98%]			
SPH-SW09- 14	JF917178	506	*Alphaproteobacteria[100%]*	99%	EU409478	*Lake Grosse Fuchskuhle isolate* Fuku2-ISO-153
			Rhodospirillales[99%] Rhodospirillaceae[90%]	94%	GU25643	*Azospirillum amazonense* DSM 2787^T
			Rhodocista[38%]			
SPH-SW09-15	JF917179	530	*Gammaproteobacteria[100%]*	95%	AJ290055	Lake Grosse Fuchskuhle clone FukuN13
			Methylococcales[100%]			
			Methylococcaceae[100%] Methylobacter[100%]			

SPH-SW09-16	JF917180	506	*Alphaproteobacteria[100%] Sphingomonadales[100%] Sphingomonadaceae[100%] Novosphingobium[99%]*	99%	EU336977	*N. acidiphilum* FSW06-204d^T
SPH-SW09-17	JF917181	506	*Alphaproteobacteria[100%] Sphingomonadales[100%] Sphingomonadaceae[100%] Novosphingobium[99%]*	99%	EU336977	*N. acidiphilum* FSW06-204d^T
SPH-SW09-18	JF917182	506	*Alphaproteobacteria[100%] Rhodospirillales[99%] Rhodospirillaceae[92%] Rhodocista[30%]*	99%<br>94%	EU409478<br>GU256437	Lake Grosse Fuchskuhle isolate Fuku2-ISO-153<br>*Azospirillum amazonense* DSM 2787^T

Reference

1. Wang Q, Garrity GM, Tiedje JM, Cole JR (2007) Naive Bayesian classifier for rapid assignment of rRNA sequences into the new bacterial taxonomy. Appl Environ Microbiol 73: 5261-5267.
2. Altschul SF, Gish W, Miller W, Myers EW, Lipman DJ (1990) Basic local alignment search tool. J Mol Biol 215: 403-410.
