# Supplementary material for: Contrasting Effects of Singlet Oxygen and Hydrogen Peroxide on Bacterial Community Composition in a Humic Lake
Source: PLoS One. 2014 Mar 25;9(3):e92518. doi: 10.1371/journal.pone.0092518 (PMC3965437; doi:10.1371/journal.pone.0092518)
Supplement: Materials S1 — Characterization of water sample photo-reactivity, including NOM characteristics of south-west compartment samples, comparison of water sample photo-reactivity, in situ H2O2 formation and decay, and the potential photochemical effects of unbleached material from the acidic fen area. Investigation of bacterial community composition by 16S rRNA (gene) based methods by the generation and screening of 16S rRNA gene clone libraries, 16S rRNA targeting reverse transcriptase (RT)-PCR DGGE analysis and group-specific 16S rRNA targeting RT-PCR DGGE analysis. (PDF) [file pone.0092518.s016.pdf]

Supporting material

Contrasting effects of singlet oxygen and hydrogen peroxide on bacterial community composition in a humic lake

Stefanie P. Glaeser, Bork A. Berghoff, Verena Stratman, Hans-Peter Grossart, and Jens Glaeser

Supplementary Methods and Results

Characterization of water sample photo-reactivity

NOM characteristics of SW basin samples

The chemical characteristics of natural organic matter (NOM) in a lake largely depend on the trophic status and the catchment area features. NOM characteristics change over time and during seasons, which may strongly affect bacterial community composition (BCC). Especially in the upper, illuminated water layers, photochemical reactions generate toxic compounds including reactive oxygen species (ROS) [1,2,3,4]. In order to compare NOM concentrations and reactivity between experimental periods, water samples were obtained on 2nd July 2006, 5th September 2008, and 14th August 2009.

The reactivity of NOM between experiments was compared by using 0.22 µm filtered lake water samples that were stored at -20°C directly after sampling. Values for the specific UVA absorbance at 254 nm ($SUVA_{254}$) were determined according to [5] by using a Specord

50 UV-VIS spectral photometer (Analytic Jena, Germany). Total organic carbon (TOC) analysis was performed with a LiquiTOC DOC Analyser (Elementar Analysensysteme, Germany). NOM concentrations were different on the respective experiment days as indicated by values of 23.3, 34.0, and 28.4 mg C L^{-1} in 2006, 2008, and 2009 (Table S1). Respective $SUVA_{254}$ values of 3.3, 3.7, and 4.3 reflect the specific UV absorbance of NOM at 254 nm and indicate that chemical signature was different between samples (Table S1). Hence reactivity of SW basin water samples may have changed between experiments. Since a direct correlation of $SUVA_{254}$ values, which indicates the specific UV absorbance and photo-reactivity could not be established [5], formation of $^1O_2$ and H_2O_2 were determined experimentally.

Comparison of water sample photo-reactivity

A direct comparison of ROS formation by artificial irradiation of NOM in water samples was performed with 50 W m^{-2} of UV-A and 1 W m^{-2} UV-B (Helarium B1-12-40W, Wolff System, Stuttgart, Germany). This UV light intensity was ~2-fold higher than the maximum natural UV radiation on a bright summer day at the lake site. UV-A and UV-B light intensities were measured by using respective UV Sensors (Type 2.3 and Type 1.3 for UV-A and UV-B, respectively, Indium Sensors, Neuenhagen, Germany) and a data-logger (LI-1400, LI-COR, Bad Homburg, Germany). Singlet oxygen steady state concentrations were determined according to the method of [6] as described recently [7]. For H_2O_2 concentrations the method described by [8] was used with slight modifications: 300 µL of 0.22 µm filtered water samples were mixed with 9 µL freshly prepared Amplex Red Assay consisting of 100 µL distilled water and 200 µL Horseradish peroxidase (Sigma Aldrich, Seelze, Hannover; 50 U mL^{-1} in 250 mM Tris-HCl, pH7.2), and 2.5 µL Amplex Red (Sigma Aldrich, 1.25 mg mL^{-1} in DMSO). Fluorescence was measured in a SFM 25 fluorimeter (Kontron, Eiching, Germany) with excitation set to 570 nm and emission to 585 nm. Alternatively, the Infinite 200 microtiter plate

reader (Tecan, Männedorf, Switzerland) was used. Blanks were prepared by adding 10 U of catalase (Sigma Aldrich).

Singlet oxygen steady state concentrations were increased by 1.12 and 1.24-fold in samples from 2008 and 2009 compared to that from 2006. In a similar manner, H_2O_2 formation increased by 1.5 and 4.5-fold, respectively (Table S1). Normalization to DOC concentrations showed that $[^1O_2]_{SS}$ ranged between 0.55 and 0.73 × 10^{-14} M per mg DOC L^{-1}. In contrast, the rate of H_2O_2 formation increased from 0.23 to 1.04 µM per mg DOC L^{-1} (Table S1). Changes in $^1O_2$ and H_2O_2 formation were not correlated. Therefore, we conclude that different chemical signatures of NOM may have caused differences in $^1O_2$ and H_2O_2 formation. Recently published experiments also indicate a limited correlation between $^1O_2$ and H_2O_2 formation from DOM [9]. The formation of $^1O_2$ was not affected by the addition of 10 µM H_2O_2 to 0.22 µm filtered SW basin water samples in 2006 (data not shown). The ratio of H_2O_2 generation rate and $[^1O_2]_{SS}$ determined from UV irradiation experiments was similar in 2006 and 2008, but increased by 2.7 to 3.6-fold in 2009 (Table S1). This clearly indicates that the rate of formation of individual ROS largely depends on the time of sampling and the chemical signature of the NOM present in the lake.

In situ H_2O_2 formation and decay

In situ H_2O_2 formation and decay deserved more attention, because H_2O_2 concentrations were low compared to similar boreal lakes [10]. In 2006, a decay of 0.68 and 0.28 µM H_2O_2 h^{-1} was observed for unfiltered and 0.22 µm filtered surface water samples, respectively. In order to analyse abiotic *in situ* H_2O_2 formation rates, 0.22 µm filtered water samples with completely decayed H_2O_2 were used. In these samples, a rate of 0.44 µM H_2O_2 h^{-1} was observed during incubation in the surface water layer at ~800 W m^{-2} of sunlight intensity. These findings indicate that H_2O_2 generation and degradation is nearly balanced in the SW basin surface water.

The use of fresh water samples in 2008 further revealed a rather high H_2O_2 degradation capacity even in the absence of bacteria. Water samples were 0.22 µm filtered directly after sampling and immediately exposed to sunlight in Whirl-Pak bags. Formation of H_2O_2 was only detected at a light dose of >300 to 400 W m^{-2} (Fig. S1). This indicates a high H_2O_2 degradation capacity which is not directly linked to the activity of bacterial cells.

Potential photochemical effects of unbleached material from the acidic fen area

Reactivity of NOM in the SW basin is strongly affected by the entry of unbleached NOM from the adjacent fen located in the catchment area next to the SW basin of Lake Grosse Fuchskuhle [11]. To test this hypothesis, ROS formation was investigated in 0.22 µm filtered water samples obtained from plastic tubes inserted in the fen area [12]. This unbleached NOM generated 2 to 8-fold more H_2O_2 per mg DOC upon UV-A/B irradiation but similar $[^1O_2]_{SS}$ concentrations were observed (Table S1). Our results indicate that changes of NOM characteristics due to the entry of unbleached NOM from the fen area strongly affect H_2O_2 formation, but only weakly that of $[^1O_2]_{SS}$ concentrations. Diurnal values for rainfall and global radiation were plotted for 30 days preceding the respective experiments of 2006, 2008, and 2009 (Fig. S9). However, kinetics of rainfall and global radiation and also the total cumulative amounts of rainfall and radiation for up to 30 days preceding the experiments cannot explain differences in NOM reactivity. Hence, the impact of increased run-off of NOM from the adjacent fen into the SW basin or, in contrast, the prolonged duration of NOM bleaching on the formation of $^1O_2$ and H_2O_2 in the SW basin needs to be assessed in greater detail in the future.

Investigation of bacterial community composition by 16S rRNA (gene) based methods

Differences in experimental procedures

The experimental set ups varied slightly between the individual experiments. However, in all three experiments the same controls were performed and all data are expressed with reference

to the internal controls. The internal controls included dark incubations of water samples with and without the $^1O_2$ photosensitizer Rose Bengal and addition of H_2O_2 solution, as well as light exposure without addition of ROS generating chemicals.

The pore size of filter membranes had to be changed after a pilot phase in 2005 and 2006 for technical reasons: serial filtration by using a 5µm pore size syringe disk filter connected to a Sterivex cartirige with 0.2 µm pore size saved valuable time for sampling during the experiments. Microscopic inspection of the samples by filtation on 0.22 µm membrane filtes followed by Sybr Green I staining, indicated that free-living bacteria were smaller than 1 µm and that particles were mostly larger than 20 µm. Therefore, the same particles should have been reliably separated from the free-living size fraction irrespective whether filter pore size was 8 or 5 µm for serial fitration. This is supported by the very similar DGGE pattern of the free-living fraction in 2006, 2008, and 2009 (Fig. 10 and 11). Also, in all three experiments DGGE patterns of the particle attached fraction clearly differd from those of the free-living fraction (Fig. 10). Thererfore, we are confident that the slight changes in filter pore sizes did not significantly affect the fractionation in our study.

Exclusion of UV light was performed in a larger experimental set up to verifiy the previous finding that UV light does not stongly affect formation of ROS in Lake Grosse Fuchskuhle. UV light is absorbed within the first few cm in the water column and does not reach the Whirl-Pak bags incubated at 10 cm (data not shown). The transmission spectra for the Whirl-Pak bags are shown in Fig. S7, which indicate that PP and PE bags do not severely absorbed UV light and that the UV block sheet screend UVA and –B light, but no visible light.

Because all controls were performed for each experiment and relative changes in $^1O_2$ and H_2O_2 were analyzed with reference to the controls, slight variations in the experimental set up are considered by the overall experimental design.

Generation and screening of 16S rRNA gene clone libraries

Nearly full-length 16S rRNA gene clone libraries were generated for selected treatments with *Bacteria* primers 8F (5′-AGAGTTTGATCMTGGCTCAG-3′) and 1492R (5′-GGYTACCTTGTTACGACTT-3′) [13]. PCRs were performed in triplicates in total volumes of 20 µl containing 2 µL 1:10 diluted DNA, 1 × PCR-Buffer, 400 µM dNTPs-Mix (Fermentas, St. Leon-Rot, Germany), 0.5 µM of each primer, 0.4 µg μL^{-1} BSA (Fermentas), and 0.02 U μL^{-1} *Taq* DNA polymerase (Qiagen, Hilden, Germany). BSA was added to minimize the inhibition by co-extracted humic substances. PCR amplifications were performed in a Primus 96 Plus cycler (MWG, Ebersberg, Germany) as followed: 95°C for 3 min, 32 cycles of 94°C for 45 sec, 54°C for 45 sec, and 68°C for 1 min 30 sec, and finally 68°C for 30 min to increase the T-overhang for TA-cloning. PCRs controlled by agarose-gelelectrophoresis were pooled and purified using a PCR purification kit (Qiagen) and cloned using the Qiagen PCR cloning kit as described by the manufacturer (Qiagen). Screening for positive clones was done by colony-PCR using plasmid primers M13R (5′-AACAGCTATGACCATG-3′) and M13F (-20) (5′-GTAAAACGACGGCCAGT-3′) in a total volume of 10 µL including a colony of a clone, 1 × PCR-buffer (Molzym, Bremen, Germany), 400 µM dNTP-mix, 0.4 µM of each primer, 0.4 µg μL^{-1} BSA, and 0.02 U μL^{-1} Mol *Taq* DNA Polymerase (Molzym). PCR cycling conditions were: 95°C for 3 min, 28 cycles of 94°C for 30 sec, 55°C for 30 sec, and 68°C for 1 min 50 sec, and finally 68°C for 15 min. PCR products were controlled by agarose-gelelectrophoresis and PCR with positive inserts were re-amplified by Nested-PCR with cloning primers 8F and 1492R in a total volume of 15 µL including 0.1 µL colony-PCR. PCR and cycling conditions were slightly different as described above: without BSA, an annealing temperature of 54°C, and a primer extension time of 1 min and 30 sec. Thereafter, PCR products were controlled by agarose-gelelectrophoresis and 5 µl of the PCR were either digested with the restriction enzymes HaeIII or RsaI (Fermentas) in a total volume of 10 µl containing 0.1 U restriction enzyme and 1× restriction buffer. Samples were digested for 3 h at 37°C. PCR amplifications

and restriction digestions were performed in a C1000™ Thermal Cycler (Bio-Rad, Munich, Germany) in 96-well microtiter plates. Restriction products were separated in 1.5% agarose gels in 1× TBE buffer for 2.5 h at 3.8 V cm^{-1}. For comparison, the 100 bp DNA ladder plus (Fermentas) was loaded three times per gel. EtBr-stained gels were analysed with Gel Compare II version 4.5 (Applied Maths, Sint-Martens-Latem, Belgium) using the unweighted pair group method using arithmetic average (UPGMA) clustering based on the band-based Dice similarity matrix. Operational taxonomic units (OTUs) were defined as the combination of the restriction pattern of HaeIII and RsaI. Approximately 50 clones were investigated for each clone library. Representatives of all OTUs, which occurred more than once in a clone library, were sequenced by the Sanger method from colony-PCRs. Most abundant OTUs were sequenced with plasmid primers from the 5′and 3′ ends, whereas less abundant OTUs were only sequenced with the cloning primer 8F. Furthermore, most abundant OTUs were sequenced from different treatments and years to ensure the sequence identity within an OTU. Sequences were processed manually based on electropherograms using MEGA 5 [14]. A first phylogenetic assignment was done using the RDP II Classifier [15] and BLAST [16]. Chimera checks were performed using the Pintail software [17] and all sequences (and as a consequence respective OTUs) with low pintail values were excluded from the analysis. The coverage of each clone library was determined with the equation $C = (1-n/N) \times 100$, where N represents the number of investigated clones and n the number of unique OTUs [18]. Rarefaction curves were generated with Analytic Rarefaction version 2.0 (www.HuntMountainSoftware.com).

16S rRNA targeting reverse transcriptase (RT)-PCR DGGE analysis

Changes in BCC of metabolically active *Bacteria* were investigated by 16S rRNA targeting RT-PCR DGGE analysis using primer-systems targeting *Bacteria* or specific bacterial groups (see below). All used primer-systems and respective amplification conditions are listed in Table S4. RT-PCR amplifications were performed using the QIAGEN OneStep RT-PCR Kit in a total

volume of 15 µL including 1.5 µL of 1:10 diluted RNA, 5× QIAGEN OneStep RT-PCR buffer, 400 µM of each dNTP, 0.4 µM of each primer, 0.4 µg μL^{-1} BSA, and 0.6 µL of the OneStep RT-PCR Enzyme Mix. Quantification of PCR products and DGGE analysis were performed as described previously [7], except DGGE gels were stained with EtBr.

Group-specific 16S rRNA targeting RT-PCR DGGE analysis

We used group-specific RT-PCR DGGE analysis to investigate the effects of increased $[^1O_2]_{SS}$ and H_2O_2 concentrations on phylotype composition within abundant bacterial groups and focused on the investigation of *Actinobacteria*, *Betaproteobacteria*, and *Sphingomonadaceae,* the most dominant bacterial groups in the SW basin of Lake Grosse Fuchskuhle [19]. Primers used for this approach are listed in Table S4.

We tested three different *Betaproteobacteria*-specific primer-systems with respect to the amplification specificity and the separation potential of abundant *Betaproteobacteria* phylotypes on the DGGE gel: (i) Beta680F/GC-1055R, (ii) GC339F/Beta680R, and (iii) GC-Beta350F/Beta680R). *Betaproteobacteria* phylotypes observed by clone library analyses and *Betaproteobacteria* isolates of relevant groups determined in the SW basin *(Polynucleobacter*, *Limnohabitans*-related, *Herbaspirillum*) were used for the establishment of the *Betaproteobacteria*-specific primer-systems. The resolution power of the three different *Betaproteobacteria*-specific primer-systems was tested on DGGE gels after amplification specificity was optimized by gradient-PCRs. Only the primer-system Beta-359F/Beta-680R showed a clear separation of the DGGE bands representing the *Betaproteobacteria*-OTUs also of very closely related phylotypes. The primer-system showed a high specificity because all sequenced DGGE bands represented *Betaproteobacteria* (Table 1, Fig. 10). The primer-system was established by [20], where it was only used as a first PCR as template for a Nested-PCR approach for DGGE analysis with universal *Bacteria* primers.

For *Actinobacteria*-specific RT-PCR DGGE analysis we used the primer-system HGC236F/HGC664R [21] that was established for DGGE analysis by [22]. Sequence analysis of selective DGGE bands showed that DNA bands were affiliated to *Actinobacteria* and – mainly under elevated $^1O_2$ exposure – also to *Verrucomicrobia* (Fig. 9). Occasional detection of *Verrucomicrobia* has been previously observed by [22]. This unspecific amplification did not affect our results in regard to effects of $^1O_2$ and H_2O_2 on *Actinobacteria*, because we sequenced all predominant DNA bands.

At first, we also aimed to investigate changes of BCC within the *Alphaproteobacteria*. Therefore, we tried to use the primer Alf986, which is based on the FISH probe established by [23]. For DGGE analysis we combined this primer with two different *Bacteria* forward primers, GC-339F and GC-517F. The resolution of DGGE patterns was reasonably good, but sequence analysis of abundant DGGE bands showed that *Polynucleobacter* sp. (*Betaproteobacteria*) and *Actinobacteria* represented the most abundant DGGE bands next to the DGGE band representing *N. acidiphilum* (*Alphaproteobacteria*) (data not shown). Therefore, instead of investigating all *Alphaproteobacteria* we focused on the *Sphingomonadaceae*-specific RT-PCR DGGE analysis (Fig. 10). At first we aimed to use the primer-system Sphingo108F/GC-Sphingo420R established for *Sphingomonadaceae*-specific DGGE analysis by [24]. However, the reverse primer used by [24] had two mismatches in the 16S rRNA gene sequence of *N. acidiphilum*, the most abundant *Alphaproteobacterium* in the south-west basin of Lake Grosse Fuchskuhle. Therefore, we used the *Alphaproteobacteria*-specific primer ADF689R as reverse primer. Unfortunately, several *Novosphingobium* type strains did not show a clear separation of DGGE bands and resulted in up to three DGGE bands for one strain. Based on these results, we decided to use a nested-PCR approach with the *Sphingomonadaceae*-specific primer-system Sphingo108F/Alf968R for pre-amplification and the *Bacteria* primer-system GC-339F/907R for amplification for DGGE analysis. Different *Sphingomonadaceae* isolates observed from the SW basin were clearly separated by this approach (data not published). Sequence analysis of

the DGGE bands determined in the *Sphingomonadaceae*-specific RT-PCR DGGE analysis showed that the major DNA band represented *N. acidiphilum*, whereas all other sequenced DNA bands were affiliated to *Alphaproteobacteria* other than *Sphingomonadaceae*. Those unspecific DNA bands occurred only in samples where *N. acidiphilum* seemed to be reduced in abundance as observed by *Bacteria*-specific DGGE and clone library analysis. In contrast to other *Alphaproteobacteria*, these sequences represent less abundant phylotypes since they were not observed in any 16S rRNA gene clone library or by *Bacteria* RT-PCR DGGE analysis. More abundant *Alphaproteobacteria* determined by *Bacteria* based analysis were not found. Our results indicate that *N. acidiphilum* was the predominant *Sphingomonadaceae* species in the SW basin at the time when all experiments were performed. No further *Sphingomonadaceae* were detected.

References

1. Baxter RM, Carey JH (1983) Evidence for photochemical generation of superoxide ion in humic waters. Nature 306: 575-576.

2. Cooper WJ (1989) Sunlight induced photochemistry of humic substances in natural waters: major reactive species. Adv Chem Ser 219: 332-362.

3. Zepp RG, Wolfe NL, Baughman GL, Hollis RC (1977) Singlet oxygen in natural waters. Nature 267: 421-423.

4. Cooper WJ, Lean DRS (1989) Hydrogen-peroxide concentration in a northern lake - photochemical formation and diel variability. Environ Sci Technol 23: 1425-1428.

5. Weishaar JL, Aiken GR, Bergamaschi BA, Fram MS, Fujii R, et al. (2003) Evaluation of specific ultraviolet absorbance as an indicator of the chemical composition and reactivity of dissolved organic carbon. Environ Sci Technol 37: 4702-4708.

6. Haag WR, Hoigne J (1986) Singlet oxygen in surface waters. 3. Photochemical formation and steady-state concentrations in various types of waters. Environ Sci Technol 20: 341-348.

7. Glaeser SP, Grossart H-P, Glaeser J (2010) Singlet oxygen, a neglected but important environmental factor: short-term and long-term effects on bacterioplankton composition in a humic lake. Environ Microbiol 12: 3123-3136.

8. Tranvik L, Kokalj S (1998) Decreased biodegradability of algal DOC due to interactive effects of UV radiation and humic matter. Aquat Microb Ecol 14: 301-307.

9. Dalrymple RM, Carfagno AK, Sharpless CM (2010) Correlations between dissolved organic matter optical properties and quantum yields of singlet oxygen and hydrogen peroxide. Environ Sci Technol 44: 5824-5829.

10. Häkkinen PJ, Anesio AM, Granéli W (2004) Hydrogen peroxide distribution, production, and decay in boreal lakes. Can J Fish Aqua Sci 61: 1520-1527.

11. Sachse A, Babenzien D, Ginzel G, Gelbrecht J, Steinberg CEW (2001) Characterization of dissolved organic carbon (DOC) in a dystrophic lake and an adjacent fen. Biochemistry 54: 279-296.

12. Buck U, Babenzien HD, Zwirnmann E (2008) Extracellular peroxidase activity in an experimentally divided lake (Grosse Fuchskuhle, northern Germany). Aquat Microb Ecol 51: 97-103.

13. Lane DJ (1991) 16S/23S rRNA sequencing. In: Stackebrandt E, Goodfellow M, editors. Nucleic acid techniques in bacterial systematics. Chichester: John Wiley. pp. 115-174.

14. Tamura K, Peterson D, Peterson N, Stecher G, Nei M, et al. (2011) MEGA5: Molecular Evolutionary Genetics Analysis Using Maximum Likelihood, Evolutionary Distance, and Maximum Parsimony Methods. Mol Biol Evol 28: 2731-2739.

15. Wang Q, Garrity GM, Tiedje JM, Cole JR (2007) Naive Bayesian classifier for rapid assignment of rRNA sequences into the new bacterial taxonomy. Appl Environ Microbiol 73: 5261-5267.

16. Altschul SF, Gish W, Miller W, Myers EW, Lipman DJ (1990) Basic local alignment search tool. J Mol Biol 215: 403-410.

17. Ashelford KE, Chuzhanova NA, Fry JC, Jones AJ, Weightman AJ (2005) At least 1 in 20 16S rRNA sequence records currently held in public repositories is estimated to contain substantial anomalies. Appl Environ Microbiol 71: 7724-7736.

18. Wilson JB (1991) Methods for fitting dominance diversity curves. J Veg Sci 2: 35-46.

19. Allgaier M, Grossart H-P (2006) Diversity and seasonal dynamics of *Actinobacteria* populations in four lakes in northeastern Germany. Appl Environ Microbiol 72: 3489-3497.

20. Mühling M, Woolven-Allen J, Murrell JC, Joint I (2008) Improved group-specific PCR primers for denaturing gradient gel electrophoresis analysis of the genetic diversity of complex microbial communities. ISME J 2: 379-392.

21. Glöckner FO, Zaichikov E, Belkova N, Denissova L, Pernthaler J, et al. (2000) Comparative 16S rRNA analysis of lake bacterioplankton reveals globally distributed phylogenetic clusters including an abundant group of *Actinobacteria*. Appl Environ Microbiol 66: 5053-5065.

22. Allgaier M, Bruckner S, Jaspers E, Grossart HP (2007) Intra- and inter-lake variability of free-living and particle-associated *Actinobacteria* communities. Environ Microbiol 9: 2728-2741.

23. Neef, A (1997) Anwendung der *in situ*-Einzelzell-identifizierung von Bakterien zur Populationsanalyse in komplexen mikrobiellen Biozönosen. Dissertation Technical University of Munich.

24. Leys NM, Ryngaert A, Bastiaens L, Top EM, Verstraete W, et al. (2005) Culture independent detection of *Sphingomonas* sp EPA 505 related strains in soils contaminated with polycyclic aromatic hydrocarbons (PAHs). Microb Ecol 49: 443-450.
